# Optimized New Shengmai Powder suppresses ferroptosis in ischemic cardiomyocytes via cGMP-PKG signalling

**DOI:** 10.1186/s13020-026-01364-6

**Published:** 2026-03-06

**Authors:** Zeyu Zhang, Zhihua Yang, Zhuangzhuang Jia, Yuwei Song, Liuli Guo, Shuai Wang, Xianliang Wang, Jingyuan Mao

**Affiliations:** 1 First Teaching Hospital of Tianjin University of Traditional Chinese Medicine, Tianjin, 300381 People’s Republic of China; 2 National Clinical Research Center for Chinese Medicine, Tianjin, China; 3 Tianjin University of Traditional Chinese Medicine, Tianjin, 301617 China; 4 School of Basic Medical Sciences, Yunnan University of Chinese Medicine, Kunming, 650500 China

**Keywords:** Ischemic heart failure, Optimized New Shengmai Powder, Oxidative stress, Ferroptosis, CGMP-PKG pathway

## Abstract

**Background:**

Shengmai Powder is a classic Traditional Chinese Medicine formula that has been used for centuries to manage cardiovascular diseases characterized by “Qi–Yin deficiency with blood stasis.” Optimized New Shengmai Powder (ONSMP), a modern refinement of this formula, has shown clinical efficacy in improving cardiac function in patients with ischemic heart failure. Although our previous studies have demonstrated pronounced cardioprotective effects of ONSMP in preclinical models, the underlying molecular mechanisms—particularly those regulating cardiomyocyte death—remain incompletely understood at the cellular level. Clarifying these mechanisms is essential to bridge traditional therapeutic practice with contemporary scientific validation and to substantiate the clinical utility of ONSMP with modern mechanistic evidence.

**Purpose:**

This study elucidates the molecular mechanisms by which ONSMP inhibits ferroptosis in cardiomyocytes.

**Methods:**

LC–MS/MS was used to identify active constituents absorbed into the systemic circulation following ONSMP administration in a rat model of ischemic heart failure. A network pharmacology approach was then applied to predict potential therapeutic targets and signaling pathways through which ONSMP may treat ischemic heart failure and inhibit cardiomyocyte ferroptosis, followed by molecular docking and molecular dynamics simulations to evaluate the binding stability between representative compounds and core targets. An oxygen–glucose deprivation (OGD) injury model was established in H9C2 cardiomyocytes to mimic the pathological microenvironment of ischemic heart failure, and cells were treated with ONSMP-containing serum in the presence or absence of core-target inhibitors. Cell viability, iron homeostasis, lipid peroxidation, mitochondrial morphology and function, and the expression of ferroptosis-related genes and proteins were assessed. Finally, the functional contributions of key active constituents to the amelioration of ischemic myocardial injury were further validated.

**Results:**

A total of 44 absorbed constituents of ONSMP were detected in rat serum. Network pharmacology analysis indicated that ONSMP targets were significantly enriched in pathways related to oxidative stress responses and lipid peroxidation, with the cGMP–PKG signaling pathway emerging as a central hub. Molecular simulations confirmed stable interactions among AKT1, eNOS, PKG1, STAT3, and GPX4. In OGD-injured H9C2 cardiomyocytes, ONSMP-containing serum significantly improved cell viability, reduced intracellular iron accumulation and malondialdehyde levels, preserved mitochondrial integrity, and upregulated GPX4 expression. These effects were associated with activation of the AKT1/cGMP–PKG axis and were partially abrogated by AKT1 inhibition. Baicalin and Kaempferide were identified as representative bioactive constituents that may contribute to these anti-ferroptotic effects.

**Conclusion:**

ONSMP exerts cardioprotective effects through multiple constituents acting on multiple targets. These effects are mediated, at least in part, by modulation of the cGMP–PKG signaling pathway, which enhances antioxidant defenses and suppresses ferroptosis in cardiomyocytes. Active constituents such as Baicalin and Kaempferide together provide a modern biological basis for the traditional therapeutic efficacy of ONSMP in ischemic heart failure.

**Supplementary Information:**

The online version contains supplementary material available at 10.1186/s13020-026-01364-6.

## Introduction

Heart failure is a complex clinical syndrome arising from structural and/or functional cardiac abnormalities and represents the terminal stage of many cardiovascular diseases [1]. Globally, more than 56.5 million people are living with heart failure, and among hospitalized patients the median survival is only 2.1 years; the 1-, 2-, 5-, and 10-year survival rates after diagnosis decline to 87%, 73%, 57%, and 35%, respectively [2–4]. In China, data from the Global Burden of Disease 2023 study indicate that approximately 14.3 million individuals had heart failure in 2023, corresponding to a crude prevalence of about 998 and an age-standardized prevalence of 677 per 100,000 population—more than one-quarter of the global burden—and confirming heart failure as a major chronic disease requiring urgent public health action [5]. Ischemic heart disease is the leading cause of heart failure, accounting for around 26.5% of cases worldwide [6] and 49.4% among hospitalized patients with heart failure in China [7]. Clinical follow-up studies show that 16.7% of patients are readmitted for worsening heart failure within 30 days after myocardial infarction, and between 12 and 22 months after the index event the risk of ischemic heart failure–related death or readmission remains high at 12%–17% [8, 9]. Despite advances in contemporary Western therapies—including pharmacological treatment, thrombolysis, revascularization, and neurohormonal blockade—that have improved myocardial perfusion, relieved symptoms, and enhanced quality of life, the overall prognosis of patients with ischemic heart failure remains unsatisfactory[10, 11]. Taken together, these findings underscore the need to move beyond traditional treatment paradigms and to develop more effective intervention strategies for heart failure.

Ischemic heart failure arises when, following regional myocardial necrosis caused by myocardial infarction, the surviving non-infarcted myocardium is subjected to chronic activation of the neuroendocrine and renin–angiotensin–aldosterone systems, hypoperfusion due to residual coronary artery lesions, and persistent oxygen supply–demand imbalance, resulting in sustained hypoxic stress and progressive pathological remodeling characterized by cardiomyocyte loss, fibrosis, and compensatory hypertrophy[12–15]. Recent studies have shown that ferroptosis, an iron-dependent form of programmed cell death driven by lipid peroxidation, plays a critical role in this process. Since Wang Fudi et al.[16] first implicated ferroptosis in cardiotoxicity and ischemia–reperfusion injury models in 2019, accumulating evidence has demonstrated its involvement across the spectrum of coronary atherosclerosis, myocardial infarction, and ischemia–reperfusion injury, where it promotes heart failure progression by influencing cardiomyocyte survival, inflammatory responses, and fibrotic remodeling [17]. Mechanistically, hypoxic stress depletes intracellular NADPH and triggers excessive ROS production, suppresses the cystine/glutamate antiporter, and leads to glutathione depletion and inactivation of the antioxidant enzyme GPX4; in parallel, ferritinophagy-mediated ferritin degradation increases the labile iron pool, while upregulation of acyl-CoA synthetase long-chain family member 4 enhances membrane polyunsaturated phospholipid content. The combined effects of impaired antioxidant defenses, iron overload, and heightened susceptibility to lipid peroxidation drive irreversible accumulation of lipid peroxidation products, ultimately inducing cardiomyocyte ferroptosis [18–20]. Thus, targeted inhibition of cardiomyocyte ferroptosis has emerged as a promising therapeutic strategy for ischemic heart failure. Clarifying the relevant signaling networks and developing preventive and therapeutic agents that modulate ferroptosis are of substantial scientific and translational interest and may open new avenues for preserving cardiac function and preventing or treating ischemic heart failure.

TCM has become an important component of mainstream clinical practice in China for the management of ischemic heart failure, owing to its favorable efficacy–toxicity profile and an increasingly solid evidence base [21]. In TCM theory, this condition is categorized under “heart failure” and frequently overlaps with the pattern of “chest impediment”; epidemiological studies have shown that its core pattern is dual qi-and-yin deficiency accompanied by blood stasis [18]. Classical texts have long described this pathophysiology. The *Huangdi Neijing* states that “when the hand Shaoyin qi is exhausted, the vessels are obstructed; when the vessels are obstructed, blood cannot flow,” highlighting the role of heart-qi deficiency in the development of blood stasis. The *Xuezheng Lun* further notes that “when blood stasis persists, its fluids accumulate; stasis transforming into fluid also leads to edema,” clarifying the progression from blood stasis to water retention. The *Jinkui Yaolue·Shuiqi Disease* chapter records that “in heart water disorder, the body feels heavy and qi is deficient, lying down is difficult, with vexation and restlessness, and yin edema develops,” which closely parallels the typical clinical manifestations of heart failure, including systemic congestion, lower-limb edema, and orthopnea. Recent advances in molecular cardiology have identified ferroptosis—an iron-dependent, lipid peroxidation-driven form of regulated cell death—as a critical mechanism in ischemic myocardial injury and cardiac remodeling. Notably, TCM interprets the pathological basis of ferroptosis in heart failure within the framework of “qi deficiency with blood stasis and mutual congealing of phlegm and stasis.” Qi deficiency weakens the driving force of blood, leading to circulatory stasis and vascular obstruction, while loss of yang-warming function permits blood to congeal and combine with pathological fluids to form phlegm–stasis complexes. At the cellular level, qi deficiency corresponds to mitochondrial dysfunction, impaired energy metabolism, and collapse of endogenous antioxidant defenses (e.g., GPX4), whereas blood stasis reflects chronic low-grade inflammation and microcirculatory insufficiency—conditions that promote iron accumulation and uncontrolled lipid peroxidation, thereby triggering ferroptosis. This conceptual alignment provides a mechanistic bridge between TCM pattern differentiation and modern cell death pathways [18].

Drawing on extensive clinical experience, Professor Mao Jingyuan, a National Famous Doctor of Chinese Medicine and Qihuang Scholar, developed ONSMP from the classical formula *Shengmai San*, in line with the core therapeutic principle in traditional Chinese medicine of “tonifying qi, activating blood, and promoting diuresis.” ONSMP is specifically formulated for heart failure characterized by the pattern of dual qi-and-yin deficiency with blood stasis. Clinical studies have shown that ONSMP not only significantly alleviates key symptoms such as palpitations, dyspnea, and edema, but also improves exercise tolerance, upgrades NYHA functional class, reduces rehospitalization, and enhances long-term quality of life, with a favorable safety profile [22]. In a rat model of post–myocardial infarction heart failure, our previous work demonstrated that ONSMP significantly improved cardiac function, reduced myocardial fibrosis, and attenuated ventricular remodeling. Mechanistic investigations further revealed that ONSMP confers cardioprotection at least in part by modulating cardiomyocyte ferroptosis and enhancing myocardial antioxidant defenses, thereby providing experimental support for the clinical efficacy of this traditional formula [23].

Given the complex composition and multi-target nature of ONSMP, its mechanisms in inhibiting cardiomyocyte ferroptosis remain incompletely understood. Moreover, conventional animal experiments are limited by the cellular heterogeneity of myocardial tissue, which reduces the precision of mechanistic inference. In this study, LC–MS/MS was first used to identify the serum-absorbed constituents of ONSMP and predict their potential targets. Network pharmacology was then integrated to elucidate the molecular pathways through which ONSMP may suppress cardiomyocyte ferroptosis in ischemic heart failure, and key molecular interactions were further evaluated by molecular docking and molecular dynamics simulations. On this basis, the anti-ferroptotic effects and underlying mechanisms of ONSMP were validated in an OGD-induced H9c2 cardiomyocyte model using ONSMP-containing serum in combination with specific inhibitors, and representative active constituents of ONSMP were subsequently selected for targeted verification. By constructing a multi-level chain of evidence that spans component identification, in silico prediction, and in vitro experimentation, this study aims to comprehensively elucidate the molecular mechanisms by which ONSMP inhibits cardiomyocyte ferroptosis in ischemic heart failure, thereby providing a robust scientific basis for its clinical application.

## Materials and methods

### Preparation of ONSMP

The herbal materials of ONSMP were supplied by the Pharmacy Department of the First Affiliated Hospital of Tianjin University of Traditional Chinese Medicine. Latin names of plant-derived crude drugs were standardized according to the Medicinal Plant Names Services (http://mpns.kew.org), and non-plant-derived materials were verified against the official standards of the Chinese Pharmacopoeia (Table 1). ONSMP was prepared at the Tianjin Institute of Pharmaceutical Research as follows. *Salvia miltiorrhiza* Bunge was processed separately: the dried herb was ground into a fine powder and reserved. The remaining eight herbs were decocted together three times in 10-fold volumes of water (2 h each time). The combined decoctions were filtered, concentrated to a thick extract, vacuum-dried, pulverized, and then thoroughly mixed with the *Salvia miltiorrhiza* Bunge powder.

**Table 1:** Components of ONSMP [23]

Chinese name	Latin binomial name	English name	Family	Medicinal parts	Dosage (g)	Country of use
Huangqi	*Astragalus membranaceus* (Fisch.) Bunge	Radix Astragali	Leguminosae	Dried root	10	China, Japan, and Korea
Dangshen	*Codonopsis pilosula* (Franch.) Nannf	Radix Codonopsis	Campanulacea	Dried root	10	China, Japan, and Korea
Ciwujia	*Eleutherococcus senticosus* (Rupr. & Maxim.) Maxim	Radix et Rhizoma seu Caulis Acanthopanacis Senticosi	Araliaceae	Dried root	10	China, Japan, Korea, and Russia
Danshen	*Salvia miltiorrhiza* Bunge	Radix et Rhizoma Salviae Miltiorrhizae	Labiatae	Dried root	10	China, Japan, Korea, America, and Europe
Biejia	*Trionyx sinensis* Wiegmann	Carapax Trionycis	Trionychidae	Carapace	10	China and Japan
Fuling	*Poria cocos* (Schw.) Wolf	Poria	Polyporaceae	Dried sclerotium	10	China, Japan, Korea, and Thailand
Tinglizi	*Lepidium apetalum* Willd	Semen Lepidii or Semen Descurainiae	Cruciferae	Dried ripe seed	6	Korea, Japan, and China
Maidong	*Ophiopogon japonicus* (Thunb.) Ker Gawl	Radix Ophiopogonis	Liliaceae	Dried root	10	Korea, Japan, and China
Zhiqiao	*Citrus* × *aurantium* L	Fructus Aurantii	Rutaceae	Dried fruit	6	Korea, Japan, and China

### LC–MS/MS

To characterize the constituents of ONSMP absorbed into the bloodstream, LC–MS/MS analyses were performed in parallel on the ONSMP decoction, serum from ischemic heart failure model rats, and serum from model rats after ONSMP administration. The ONSMP decoction was subjected to ethanol precipitation, followed by nitrogen evaporation to dryness, reconstitution, and filtration. Serum samples (n = 6 per group) underwent protein precipitation with pre-chilled methanol, followed by vortex mixing, ultrasonication, centrifugation, nitrogen evaporation, reconstitution in 50% methanol, and a second centrifugation to obtain the supernatant. Chromatographic separation was carried out on a SHIMADZU Nexera X2 LC-30AD system equipped with an ACQUITY UPLC HSS T3 column (2.1 × 100 mm, 1.8 µm) maintained at 40 °C, using a 0.3 mL/min flow rate and a 30-min gradient of acetonitrile and water containing 0.1% formic acid. Mass spectrometric detection was conducted on a Q Exactive Plus instrument with a HESI source operated in positive/negative ion switching mode. Source voltages, temperatures, and gas parameters were optimized. Full MS scans were acquired over an m/z range of 90–1350 at a resolution of 70,000, and data-dependent MS2 (Top10) spectra were collected at a resolution of 17,500 using stepped higher-energy collisional dissociation energies of 20, 30, and 40 eV. Raw data were processed in MSDIAL for peak alignment, retention time correction, and peak area extraction. Components were annotated using thresholds of < 0.01 Da for MS1 mass error, < 0.02 Da for MS2 mass error, and a matching score > 70%. Blood-absorbed constituents of ONSMP were finally assigned by retaining shared features between the ONSMP decoction and post-administration serum and excluding peaks present only in model serum.

### Network pharmacology

To predict the molecular mechanisms by which ONSMP inhibits cardiomyocyte ferroptosis in ischemic heart failure, we employed a multi-source bioinformatics strategy. SMILES structures of ONSMP constituents were obtained from PubChem (https://pubchem.ncbi.nlm.nih.gov). Potential targets were predicted using SwissTargetPrediction (http://www.swisstargetprediction.ch/), Super-PRED (https://prediction.charite.de/index.php), the Similarity Ensemble Approach (https://sea16.docking.org/), and TargetNet (http://targetnet.scbdd.com/home/index/). In addition, target annotations from HERB (http://herb.ac.cn) and TCMBank (https://www.tcmbank.cn/) were integrated to construct a comprehensive ONSMP constituent–target database. In parallel, disease-associated targets were systematically retrieved from GeneCards (https://www.genecards.org/), OMIM (https://omim.org/), PharmGKB (https://www.pharmgkb.org/), TTD (https://db.idrblab.net/ttd/), DrugBank (https://go.drugbank.com/), HERB (https://herb.ac.cn/), and FerrDb (https://ferrdb.biotec.ai/) using the keywords “ischemia,” “heart failure,” “cardiac failure,” and “ferroptosis,” and intersecting these datasets yielded putative disease-related targets regulated by ONSMP. GO functional and KEGG pathway enrichment analyses of the intersected targets were performed in R 4.3.2 using Bioconductor packages DOSE, clusterProfiler, enrichplot, and pathview. PubMed (https://pubmed.ncbi.nlm.nih.gov/) searches were further used to prioritize pathways relevant to ischemic heart failure and ferroptosis, while previously validated pathways were excluded to focus on candidate pathways. A protein–protein interaction network of pathway-enriched proteins was then constructed using STRING (https://string-db.org/), and multidimensional topological parameters—including betweenness centrality, closeness centrality, degree centrality, eigenvector centrality, local average connectivity, and network centrality—were calculated in Cytoscape 3.9.0. Core proteins were identified through two rounds of threshold filtering, and their roles in cardiomyocyte ferroptosis were further interpreted based on the published literature.

### Molecular docking

In this study, we established a multidimensional molecular docking evaluation framework that integrated the GRAMM server with the affinity scoring function of AutoDock Vina (binding free energy < 0 kcal/mol indicating spontaneous binding, <  −5 kcal/mol indicating effective binding, and <  −7 kcal/mol indicating stable binding), as well as the Docking Score (higher absolute value indicating a higher binding probability) and Confidence Score (> 0.5 indicating possible binding and > 0.7 indicating a high binding probability) from HDOCKlite v1.1. For protein–protein interactions, receptor and ligand three-dimensional (3D) structures were obtained from UniProt (https://www.uniprot.org) and the Protein Data Bank (PDB; https://www.rcsb.org), processed with PyMOL 2.3.0, and docked using GRAMM (https://gramm.compbio.ku.edu/), followed by interaction analysis with PDBePISA (https://www.ebi.ac.uk/pdbe/pisa/). For protein–DNA interactions, full-length protein structures were predicted using AlphaFold2, and the highest scoring sequences on the positive DNA strand were selected as putative binding sites using Jaspar-scan. High-confidence models of these regions were subsequently generated with AlphaFold3 and docked using HDOCKlite v1.1; molecular interactions were analyzed with PLIP (https://plip-tool.biotec.tu-dresden.de/plip-web/plip/index), which applies six-degree-of-freedom sampling and composite scoring functions. For protein–small-molecule interactions, ligand structures were retrieved from PubChem (https://pubchem.ncbi.nlm.nih.gov), energy-minimized with Chem3D 2020, prepared in AutoDock Tools, and docked in a semi-flexible mode using AutoDock Vina; binding modes were analyzed with PLIP. All docking results were visualized in PyMOL 2.3.0.

### Molecular dynamics simulation

In this study, molecular dynamics simulations were used to monitor biomolecular binding at the atomistic level, quantify binding free energies with explicit solvation, and analyze the kinetic and thermodynamic stability of the complexes. This approach provides an integrated computational framework for understanding biomolecular recognition, signal transduction, and drug action mechanisms. Docked complexes were prepared in YASARA 2020 by adding hydrogens, optimizing protonation states, and correcting structural inconsistencies. molecular dynamics simulations of 100 ns were performed with the AMBER14 force field under periodic boundary conditions, using a Berendsen thermostat (310 K) and a Parrinello–Rahman barostat (1 atm). The systems were solvated in a TIP3P water box with a 10 Å buffer and neutralized with counterions. Before the production runs, two-stage energy minimization (500 steps of steepest descent followed by 500 steps of conjugate gradient) was carried out to relieve structural strain. The long-timescale behavior and stability of the complexes under simulated environmental conditions were evaluated using RMSD, Rg, SASA, and RMSF.

### H9C2 cell modeling, grouping, and treatment

H9C2 cells (CL-0089, Pronsei Life Science Co., Ltd., Wuhan, Hubei, China) were routinely cultured in high-glucose DMEM supplemented with 10% FBS and 1% penicillin–streptomycin at 37 °C in a humidified CO₂ incubator (Heracell VIOS 160i, Thermo Scientific, MA, USA) under 5% CO_2_. The medium was changed every 2–3 days, and cells were passaged with 0.25% trypsin–EDTA when they reached approximately 80% confluence. For experiments, logarithmically growing cells were seeded into 96-well plates at 1 × 10^5^ cells/well. When the monolayer reached 80% confluence, control cells were maintained in complete DMEM, whereas model cells were switched to glucose-free DMEM containing 10% FBS and 1% penicillin–streptomycin and transferred to a tri-gas incubator (HERAcell VIOS 160i, Thermo Scientific, MA, USA) set to 95% N_2_, 3% O_2_, and 5% CO_2_ for 12, 24, or 48 h of hypoxic exposure. Cell viability was assessed using the CCK-8 assay (HY-K0301, MedChemExpress, NJ, USA), and the time point at which viability decreased to approximately 50% of control was selected as the optimal duration of OGD treatment.

To prepare drug-containing serum, SD rats were orally administered ONSMP (2.79 g/kg, twice daily for 3 d). Blood was collected 2 h after the final dose, and serum was separated, heat-inactivated at 56 °C, and stored at −80 °C. Blank serum was prepared in the same manner from rats gavaged with physiological saline. The effects of 5%–20% blank serum on H9c2 cell viability were first evaluated using the CCK-8 assay to determine the optimal baseline serum concentration. Drug-containing serum was then diluted across this concentration range while keeping the total serum content constant. Cells were initially assigned to blank control, OGD, and OGD plus different concentrations of ONSMP-containing serum, and CCK-8 was used to determine the optimal intervention concentration. Subsequent experiments were organized into five groups: blank control, OGD, OGD + ONSMP, OGD + inhibitor, and OGD + inhibitor + ONSMP. Two representative active constituents of ONSMP were further selected. After defining their maximal non-cytotoxic dose and optimal working concentration by CCK-8, eight groups were established: blank control, OGD, OGD + monomer A, OGD + inhibitor + monomer A, OGD + monomer B, OGD + inhibitor + monomer B, OGD + monomers (A + B), and OGD + inhibitor + monomers (A + B).

### Cell viability assessment

Cell viability was assessed using the CCK-8 assay (HY-K0301, MedChemExpress, NJ, USA), a scratch wound-healing assay, and Calcein AM/PI dual-fluorescence staining (E-CK-A354, Elabscience, Wuhan, China). For the CCK-8 assay, H9C2 cells were seeded into 96-well plates; after the indicated treatments, 10 μL of CCK-8 solution was added to each well. Cells were incubated at 37 °C in the dark for 1–4 h, and absorbance was measured at 450 nm using a microplate reader. Relative cell viability was calculated after background subtraction using blank wells. For the scratch assay, cells were grown in 6-well plates to 80%–90% confluence, and linear wounds were created with a sterile pipette tip. After washing with PBS, images were captured at 0, 12, and 24 h at the same fields of view using an inverted microscope (CKX41, Olympus Corporation, Tokyo, Japan), and wound-closure rates were quantified with ImageJ software. For Calcein AM/PI dual staining, the working solution was prepared by mixing Calcein AM, PI solution, and buffer at a 1:1:100 volume ratio. After PBS washing, 200 μL of staining solution was added to each well of a 24-well plate, followed by incubation at 37 °C for 10–30 min. Live (green fluorescence) and dead (red fluorescence) cells were then observed under an inverted fluorescence microscope (IX73, Olympus Corporation, Tokyo, Japan).

### Mitochondrial morphology and membrane potential Assessment

Mitochondrial ultrastructure was examined by transmission electron microscopy (HT 7800, HITACHI, Tokyo, Japan). When H9c2 cells reached approximately 80% confluence, they were detached with trypsin and centrifuged to obtain compact cell pellets 1–2 mm in diameter. The pellets were fixed at room temperature in standard fixative for 30 min and then stored at 4 °C. Subsequent sample processing and TEM imaging were carried out by Wuhan Savier Biotechnology Co., Ltd. Mitochondrial membrane potential was assessed using an enhanced JC-1 detection kit (C2003S, Beyotime, Shanghai, China). After washing cells with PBS, 1 mL of complete culture medium containing serum and phenol red was mixed with an equal volume of JC-1 working solution and added to the cells, followed by incubation at 37 °C in the dark for 20 min. The supernatant was then removed, and cells were gently washed twice with JC-1 buffer. Fresh complete medium (2 mL per well in a 6-well plate) was added, and JC-1 fluorescence was observed under an inverted fluorescence microscope (IX73, Olympus Corporation, Tokyo, Japan).

### ELISA, biochemical assays, and PB staining

To evaluate antioxidant capacity, iron accumulation, signaling pathway–related small molecules, sGC activity, and downstream effector indices in H9c2 cells, we used ELISA kits (T-SOD: JM-02137R2, GSH: JM-10683R2, GPX4: JM-10415R2, LPO: JM-10680R2, MDA: JM-10323R2, 4-HNE: JM-10761R2; Jingmei Biotechnology Co., Ltd., Yancheng, Jiangsu, China; cGMP: G775287-48 T/EA, sGC: R760007-48 T/EA; Macklin, Shanghai, China), biochemical assay kits (Fe^2^⁺: ADS-W-QT027, Fe^3^⁺: ADS-W-D007; Jiangsu Addison Biotechnology Co., Ltd., Yancheng, Jiangsu, China; NO: A012-1–2, Nanjing Jiancheng Bioengineering Institute, Nanjing, Jiangsu, China; total Fe: E-BC-K880-M, ROS: E-BC-K138-F, CAT: E-BC-K031-M, H₂O₂: E-BC-K102-M; Elabscience, Wuhan, Hubei, China), and a Prussian blue (PB) staining kit (G1428, Solarbio, Beijing, China). All assays were performed strictly according to the manufacturers’ instructions. ROS levels were visualized using an inverted fluorescence microscope (IX73, Olympus Corporation, Tokyo, Japan), and PB staining was examined under an upright optical microscope (Nikon Eclipse E100, Nikon, Tokyo, Japan). All other indices were measured using a multifunctional microplate reader (MULTISKAN FC, Thermo Fisher Scientific, MA, USA), and analyte levels were quantified from standard curves.

### IHC, IF, and IF co-localization assays

H9C2 cells were fixed with 4% paraformaldehyde for 20 min, permeabilized with 0.1% Triton X-100, washed with PBS, and blocked with 5% BSA at room temperature for 30 min. For IHC, samples were incubated with primary antibodies (4-HNE: bs-6313R, BIOSS, Beijing, China; COX2: AF7003, Affinity, Liyang, Jiangsu, China) in a humidified chamber at 4 °C for 12 h. After washing with PBS, HRP-conjugated secondary antibodies (PK10006, Proteintech, Wuhan, Hubei, China) were applied at room temperature for 1 h. Cells were then developed with DAB, counterstained with hematoxylin, differentiated with HCl–ethanol, dehydrated through graded ethanol, cleared in xylene, and mounted with neutral resin. Positive staining was observed and quantified using an upright optical microscope (Nikon Eclipse E100, Tokyo, Japan. For IF, cells were incubated with primary antibodies under the same conditions (sGC: AF0619; GPX4: DF6701, Affinity, Liyang, Jiangsu, China). After washing, fluorescence-labeled secondary antibodies (RGAR004, Proteintech, Wuhan, Hubei, China) were applied in the dark at room temperature for 2 h. Following PBS washes, samples were mounted with DAPI-containing anti-fade medium, and protein fluorescence intensity was observed and quantified using an upright fluorescence microscope (Nikon Eclipse EC1, Tokyo, Japan). For IF co-localization, two primary antibodies from different host species were mixed and incubated together: AKT1 (bsm-52010R, BIOSS, Beijing, China) with eNOS (bsm-33176 M, BIOSS, Beijing, China), and STAT3 (bsm-33301 M, BIOSS, Beijing, China) with PKG1 (A01708-3, Boster, Wuhan, Hubei, China). During secondary antibody incubation, the corresponding species-specific fluorescence-labeled secondary antibodies (RGAM004 and RGAR002, Proteintech, Wuhan, Hubei, China) were applied as a mixture. Protein co-localization and potential interaction were evaluated based on fluorescence intensity analysis, Pearson’s correlation coefficient R (range − 1 to 1; positive values indicate a positive correlation, and R > 0.5 suggests significant co-localization), and Manders’ tM1 coefficient (quantifying the spatial overlap of protein A with protein B).

### Western blot

H9C2 cells were washed three times with pre-chilled PBS and lysed in RIPA buffer supplemented with protease inhibitors. The lysates were collected, sonicated (3 × 10 s), and centrifuged at 12,000 rpm for 15 min at 4 °C to obtain the supernatant. Protein concentration was determined using a BCA assay, and the loading amount was adjusted to 20–40 μg protein per lane. Proteins were separated on SDS-PAGE gels (M00664, GenScript, NJ, USA) at 80 V until the bromophenol blue dye front entered the resolving gel, followed by 120 V to complete electrophoretic separation. For transfer, a PVDF membrane (IPVH00010, Millipore, Boston, MA, USA) was activated in methanol for 15 s and assembled into a transfer sandwich (sponge–filter paper–gel–PVDF membrane–filter paper–sponge) using rapid transfer buffer (P0575-10L, Bioyuntian, Shanghai, China). Protein transfer was performed at a constant current of 300 mA in an ice bath for 90 min. The PVDF membrane was then blocked at room temperature for 30 min with rapid blocking solution (P0252-100 mL, Bioyuntian, Shanghai, China) and incubated with primary antibodies (AKT1: bsm-52010R, p-AKT: bs-10996R, eNOS: bs-20609R, STAT3: bsm-52235R, p-STAT3: bsm-52210R, p-PKG1: bs-7368R, ACTB: bs-0061R, VCL: bsm-54148R, BIOSS, Beijing, China; p-eNOS: AF3247, sGC: AF0619, GPX4: DF6701, Affinity, Liyang, Jiangsu, China; PKG1: A01708-3, Boster, Wuhan, Hubei, China) at 4 °C overnight (12 h). After three washes with 1 × TBST, HRP-conjugated secondary antibodies were applied at room temperature for 1 h. Following additional washes, an ECL chemiluminescent substrate was added, and target protein bands were visualized using a chemiluminescence imaging system (ChemiScope 6100, Qinxian Scientific Instruments Co., Ltd., Shanghai, China).

### qRT-PCR

Total RNA was extracted from H9C2 cells using a total RNA extraction kit (G3640-50 T, Servicebio, Wuhan, Hubei, China). RNA concentration and purity were measured with a NanoDrop 2000 spectrophotometer, and samples were diluted to 200 ng/μL. cDNA was synthesized by reverse transcription using SweScript All-in-One RT SuperMix for qPCR (One-Step gDNA Remover) (G3337, Servicebio, Wuhan, Hubei, China). qPCR was performed on a real-time PCR system (CFX Connect, Bio-Rad, CA, USA) using 2 × Universal Blue SYBR Green qPCR Master Mix (G3326, Servicebio, Wuhan, Hubei, China) with the following cycling conditions: pre-denaturation at 95 °C for 30 s, followed by 40 cycles of denaturation at 95 °C for 15 s and annealing/extension at 60 °C for 30 s, and a final melt-curve analysis from 65 °C to 95 °C with fluorescence acquisition at 0.5 °C increments. All data were normalized to ACTB as the internal reference gene, and relative mRNA expression levels were calculated using the 2^−ΔΔCT^ method. Primer sequences are listed in Table 2.

**Table 2:** Primer sequences of the detected genes

Gene name	FORWARD	REVERSE	prodSize
*AKT1*	CGCTTCTTTGCCAACATCG	CACTGGCTGAGTAGGAGAACTGG	217
*eNOS*	ACTATGGCAACCAGCGTCCT	CCAGGTGTTTCTTGGGTAGGC	254
*GPX4*	AGGCAGGAGCCAGGAAGTAATC	ACCACGCAGCCGTTCTTATC	212
*STAT3*	ACAACCTGCGAAGAATCAAGC	CATCTGCTGCTTCTCCGTCACT	188
*PKG1*	GGTCACTGGTGTATGTCATGGAA	TCCGGGTACAGTTGTAAAGAATAGC	123
*sGCβ1* *ACTB*	ATTGTGGGCTTCAACGCTTTCT CTGTGCTATGTTGCCCTAGACTTC	CTGGCAAACCACTCACTGTCATAT GAACCGCTCATTGCCGATAGTG	181 120

### Statistical analysis

Data were analyzed using SPSS 21.0. Continuous variables with a normal distribution are presented as mean ± standard deviation, whereas non-normally distributed data are expressed as median (interquartile range). For analysis of group differences, if the assumptions of normality and homogeneity of variance were satisfied, one-way ANOVA was used, followed by Bonferroni post hoc tests for pairwise comparisons when overall significance was detected. If these assumptions were not met, the Kruskal–Wallis H test was applied for multiple-group comparisons, and significant results were further examined using the Mann–Whitney U test for pairwise comparisons. A *P* value < 0.05 was considered statistically significant.

## Results

### Analysis of serum-absorbed constituents of ONSMP

**Fig. 1:**
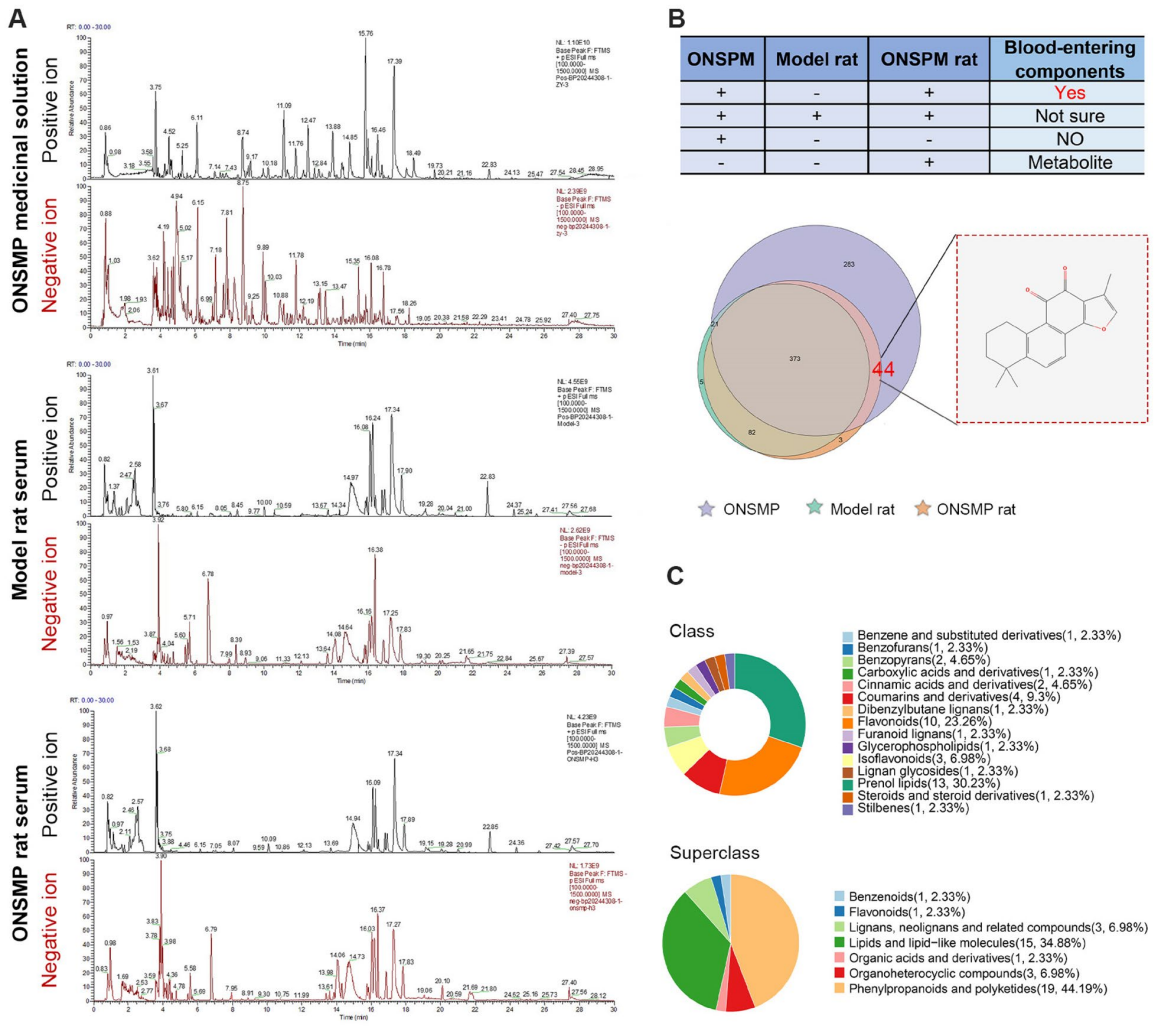
Analysis of blood-absorbed components of ONSMP. **A** Base peak chromatograms of the samples. **B** Identification of blood-absorbed components of ONSMP. **C** Classification of blood-absorbed components of ONSMP at the superclass and class levels

LC–MS/MS analysis identified 721 constituents in the ONSMP decoction, 482 compounds in serum from model rats, and 502 compounds in serum from ONSMP-treated rats (Fig. 1A, Table S1). Cross-comparison ultimately yielded 44 definitive blood-absorbed constituents of ONSMP (Fig. 1B, Table S2). These constituents were classified into seven superclasses, with phenylpropanoids and polyketides accounting for the largest proportion (44.19%), followed by lipids and lipid-like molecules (34.88%). At the class level, 15 classes were represented, predominantly prenol lipids (30.23%) and flavonoids (23.26%) (Fig. 1C).

### Prediction of the anti-ferroptotic mechanisms of ONSMP in ischemic heart failure

**Fig. 2:**
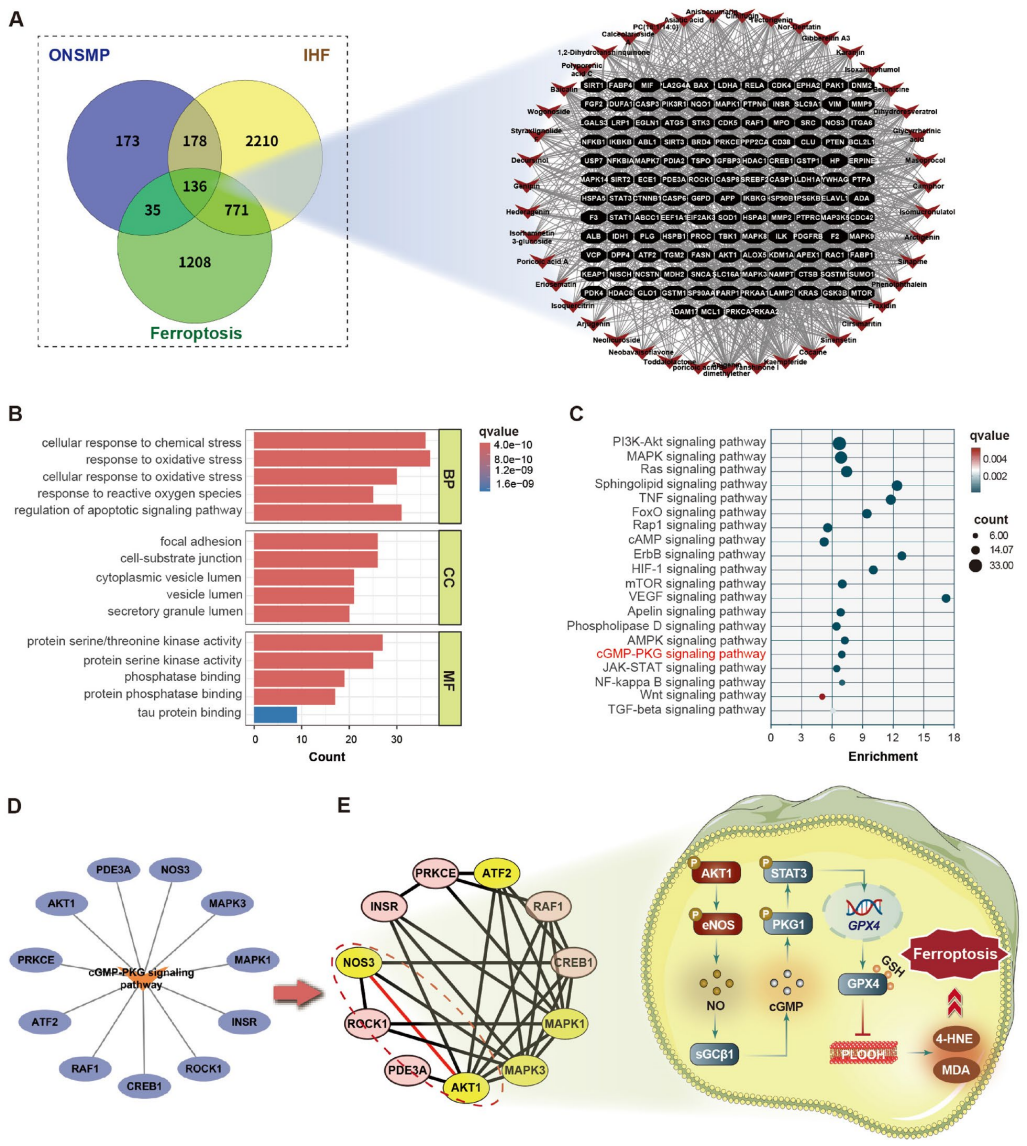
Prediction of the anti-ferroptotic mechanisms of ONSMP in cardiomyocytes. **A** Intersection of ONSMP-related targets with disease-related proteins and network topology analysis. **B** GO enrichment analysis of intersecting proteins. **C** KEGG enrichment analysis of intersecting proteins. **D** Topological analysis of the cGMP–PKG signaling pathway and its enriched proteins. **E** Identification of core pathway proteins and proposed signaling mechanism

The 44 blood-absorbed components of ONSMP were predicted to interact with 528 targets (Table S3). Intersecting these with 3295 ischemic heart failure-related proteins and 2,150 ferroptosis-related proteins yielded 136 common targets that could be regulated by 43 ONSMP components (Fig. 2A). GO enrichment analysis showed significant enrichment in biological processes such as oxidative stress response, ROS metabolism, and regulation of lipid peroxidation, highlighting core pathological mechanisms of ferroptosis. In addition, disruption of cell adhesion structures and aberrant kinase signaling were identified as further contributors to the ferroptotic process (Fig. 2B). KEGG enrichment analysis revealed multiple signaling pathways related to heart failure and ferroptosis; after excluding pathways previously validated to be regulated by ONSMP, the cGMP–PKG signaling pathway was prominently ranked and was selected as the focus of this study (Fig. 2C). A total of 11 proteins in this pathway were enriched (Fig. 2D), and PPI network analysis of these proteins identified five core nodes. Literature evidence further indicated that AKT1 and eNOS (NOS3) form an upstream regulatory module that inhibits lipid peroxidation via activation of the cGMP–PKG–GPX4 signaling axis, thereby exerting anti-ferroptotic effects in cardiomyocytes (Fig. 2E).

### Analysis of molecular interactions in the cGMP–PKG signaling pathway

**Fig. 3:**
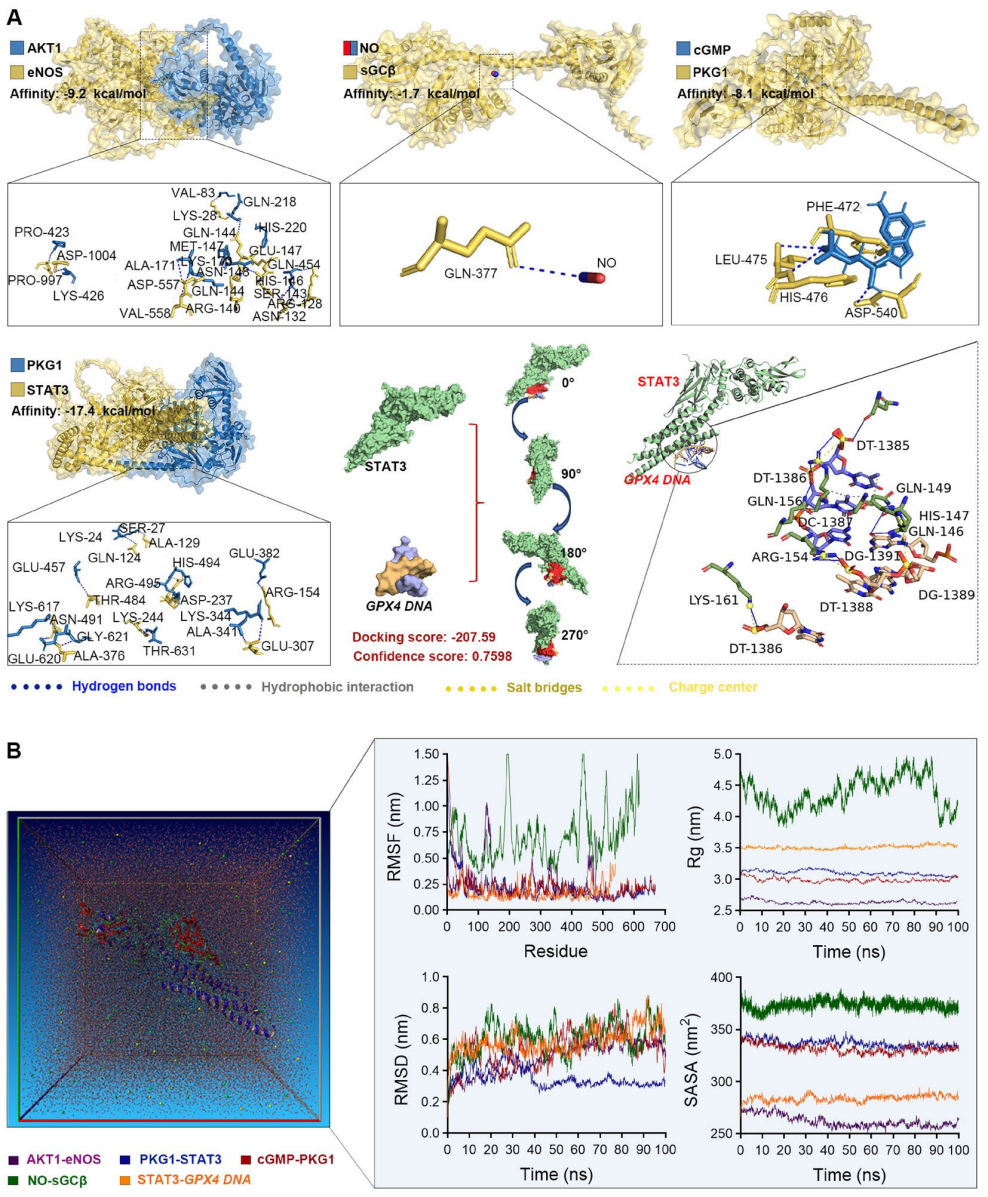
Molecular docking and dynamics of key molecules in the cGMP–PKG signaling axis. **A** Molecular docking of upstream and downstream complexes in the cGMP–PKG signaling axis. **B** Molecular dynamics simulations of these complexes

To elucidate the molecular mechanisms underlying the cGMP–PKG signaling axis, structural characterization of key nodes was performed. Docking analysis revealed strong binding at critical interfaces: the binding affinities of AKT1–eNOS, cGMP–PKG1, and PKG1–STAT3 were all less than −7 kcal/mol; the affinity of NO–sGCβ1 was less than 0 kcal/mol; and docking of STAT3 with GPX4 DNA yielded a Docking Score below − 200 and a Confidence Score above 0.7. Analysis of intermolecular interactions showed that AKT1 and eNOS were stably associated through 13 hydrogen bonds and a salt bridge between GLU-147 and HIS-220; NO specifically anchored GLN-377 of sGCβ1; cGMP formed three hydrogen bonds with PKG1 together with a hydrophobic interaction mediated by PHE-472; the PKG1–STAT3 interface comprised 10 hydrogen bonds and seven salt bridges; and STAT3 recognized *GPX4 DNA* via eight hydrogen bonds, five salt bridges, and multiple hydrophobic contacts. Taken together, this multilevel binding pattern supports efficient molecular recognition and stable complex formation among key nodes of the cGMP–PKG signaling axis (Fig. 3A).

Molecular dynamics simulations provided atomistic insights into the dynamic conformational features of key complexes within the cGMP–PKG signaling axis. The AKT1–eNOS complex exhibited a “rigid core–flexible termini” pattern: RMSF analysis showed high flexibility at residues 1–5 and 450–480 (> 0.5 nm), whereas the core region (residues 100–300) fluctuated by < 0.2 nm; Rg (2.64 ± 0.02 nm) and RMSD (0.55 ± 0.05 nm) indicated a compact conformation, and SASA (260–275 nm^2^) reflected moderate interface exposure. The NO–sGCβ1 complex achieved stable binding through large-scale rearrangements: RMSF displayed a graded distribution (N-terminal 0.3–0.6 nm, middle 1.0–1.5 nm, C-terminal 0.6–1.0 nm); the Rg trajectory initially contracted (4.5 → 3.8 nm), briefly expanded to 4.3 nm, and then converged to 3.8 nm, accompanied by continuous RMSD fluctuations (1.0–1.5 nm); SASA remained stable at 370–385 nm^2^. The cGMP–PKG1 complex demonstrated a “super-stable core”: RMSF showed a gradient (N-terminal > 0.7 nm, core < 0.3 nm), Rg rapidly converged to 2.94–3.05 nm, and RMSD increased (0.2 → 0.8 nm), indicating irreversible adjustments; SASA stabilized at 327 ± 5 nm^2^. The PKG1–STAT3 complex maintained a metastable binding state: core regions (residues 55–70 and 410–470) had RMSF values of 0.1–0.25 nm, Rg stabilized at 3.15–3.20 nm, RMSD exhibited reversible drift (mean 0.37 nm), and SASA remained at 330–345 nm^2^. The STAT3–GPX4 DNA complex combined rigidity and flexibility: the DNA-binding interface (residues 1–200) showed low RMSF (0.10–0.20 nm), whereas peripheral regions (351–541) reached 0.25–0.45 nm; Rg (3.51 ± 0.03 nm), RMSD (< 0.45 nm), and SASA (285–290 nm^2^) together maintained a functionally balanced conformation. Collectively, this multiscale analysis delineated a dynamic “rigidity–flexibility integration” interaction pattern for the cGMP–PKG signaling axis (Fig. 3B).

### Effects of ONSMP on H9C2 cell viability under OGD

#### Selection of the ONSMP-containing serum concentration and inhibitor screening

**Fig. 4:**
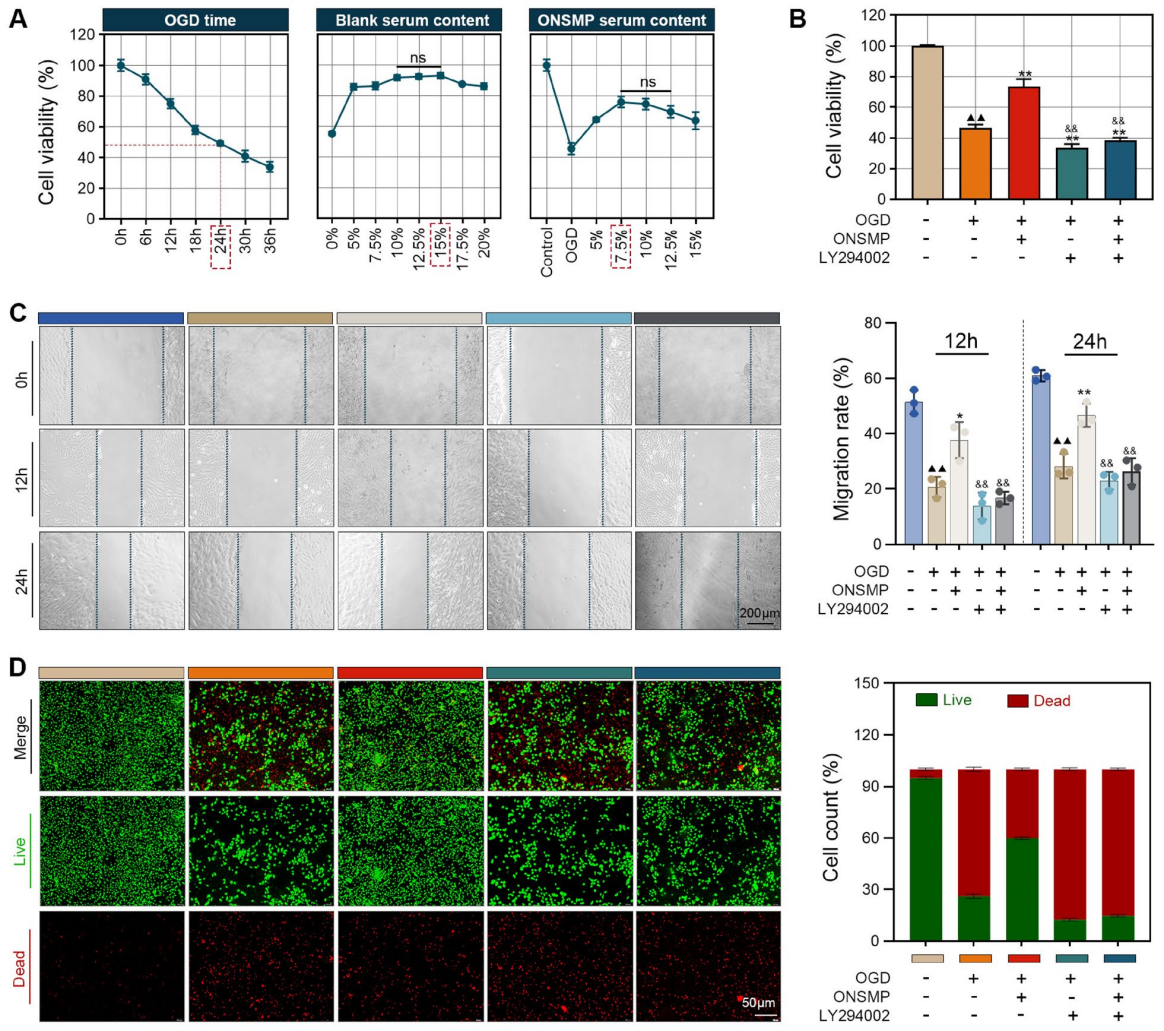
ONSMP enhances H9C2 cell viability under OGD. **A** Screening of experimental baseline conditions. **B** Assessment of cell viability. **C** Evaluation of cell migration. **D** Quantification of cell survival. (mean ± SD, n = 3). ns, not significant; ^▲▲^*P* < 0.01 vs. control group; ^*^P < 0.05, ^**^P < 0.01 vs. OGD group; ^&^*P* < 0.05, ^&&^*P* < 0.01 vs. ONSMP group

CCK-8 assays were used to establish the H9C2 OGD injury model and to determine the optimal intervention conditions for ONSMP-containing serum. Cell viability progressively decreased with prolonged OGD exposure and reached approximately 50% at 24 h; therefore, 24 h was selected as the modeling time point. The optimal concentration of drug-containing serum was then determined. Under normal culture conditions, H9C2 cell viability peaked in the presence of 10%–15% blank serum, with no significant differences among groups (*P* > 0.05), and this range was defined as the appropriate baseline culture condition. In the OGD model, with 15% total serum as the background, drug-containing serum was added in a gradient (5%–15%). Cell viability was highest in the 7.5%–15% range, with no significant differences among these groups (*P* > 0.05). Taken together, these findings indicated that 10% total serum containing 7.5% drug-containing serum was an appropriate intervention condition (Fig. 4A). On this basis, an inhibitor control system targeting AKT1—the upstream core regulatory node of the cGMP–PKG pathway—was established. According to previous reports [24, 25], cells were pretreated with the specific AKT inhibitor LY294002 (30 μM) for 30 min before OGD exposure.

#### Quality control of myocardial protein expression in rats

CCK-8 assays showed that OGD markedly reduced H9C2 cell viability, and this effect was significantly reversed by ONSMP. However, LY294002 treatment—whether administered alone or in combination with ONSMP—resulted in cell viability that was not only significantly lower than in the ONSMP group but also further decreased compared with the OGD group (Fig. 4A). Scratch assays at 12 h and 24 h demonstrated that OGD markedly inhibited cell migration, an effect that was alleviated by ONSMP, whereas LY294002-treated groups exhibited significantly lower migration rates than the ONSMP group (Fig. 4B). Quantitative live/dead staining revealed that OGD reduced the proportion of live cells from approximately 95%–26% and increased dead cells from 5 to 74%; ONSMP treatment restored the proportions of live and dead cells to 60% and 40%, respectively, whereas LY294002 treatment—with or without ONSMP—further reduced live cells to 12%–15% and increased dead cells to 85%–88% (Fig. 4C). Collectively, these findings indicate that ONSMP significantly ameliorates OGD-induced injury in H9C2 cells and that this protective effect is markedly attenuated by AKT1 inhibition.

### ONSMP alleviates OGD-induced ferroptosis in H9C2 cells

#### Reduction of ferroptosis markers and iron levels

**Fig. 5:**
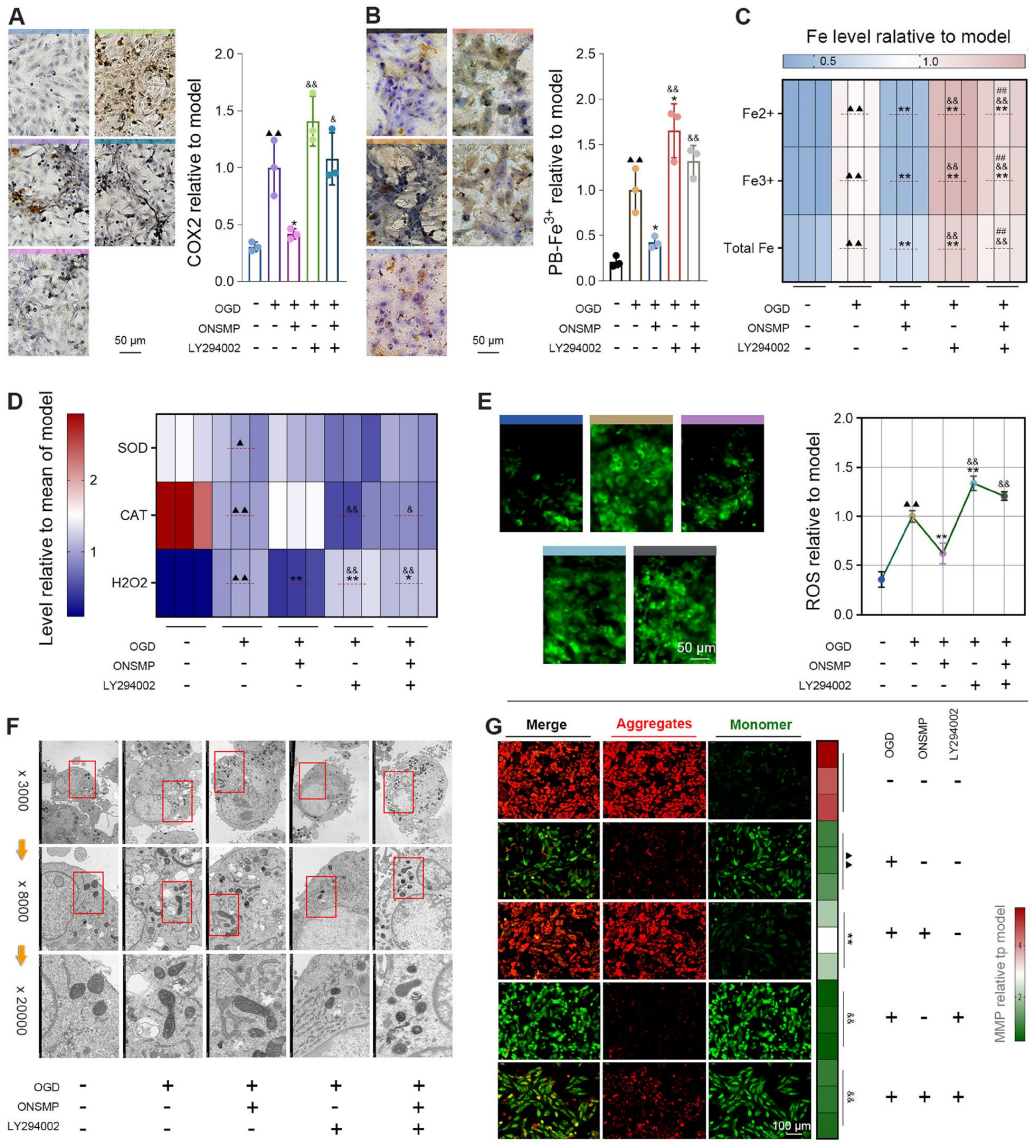
Effects of ONSMP on OGD-induced ferroptosis in H9C2 cells. **A** IHC detection of ferroptosis markers. **B** PB staining. **C** Measurement of Fe^2^⁺, Fe^3^⁺, and total Fe contents. **D** Assessment of CAT activity and SOD and H₂O₂ levels. **E** ROS level detection. **F** Transmission electron microscopy observation. **G** Mitochondrial membrane potential measurement (× 200). (mean ± SD, n = 3). ^▲^*P* < 0.05, ^▲▲^*P* < 0.01 vs. control group; ^*^P < 0.05, ^**^P < 0.01 vs. OGD group; ^&^*P* < 0.05, ^&&^*P* < 0.01 vs. ONSMP group

IHC detection of ferroptosis markers showed that COX2 expression was significantly upregulated in the OGD group compared with the control group, and this increase was effectively reduced by ONSMP treatment. In contrast, LY294002 treatment—whether administered alone or in combination with ONSMP—resulted in COX2 expression that was not only significantly higher than in the ONSMP group but also showed an increasing trend compared with the OGD group (Fig. 5A). PB staining demonstrated that iron deposition was markedly enhanced in the OGD group relative to the control group, whereas ONSMP effectively attenuated this increase. Iron deposition in the LY294002-treated groups was significantly higher than in the ONSMP group and was further increased compared with the OGD group (Fig. 5B). Consistently, quantitative measurements using assay kits showed that Fe^2^⁺, Fe^3^⁺, and total Fe contents were significantly elevated in the OGD group compared with the control group, and ONSMP treatment effectively reversed OGD-induced iron overload. In the LY294002-treated groups, Fe^2^⁺, Fe^3^⁺, and total Fe levels were all significantly higher than in the ONSMP group, and total Fe in the LY294002-only group was also significantly higher than in the OGD group. Notably, Fe^2^⁺, Fe^3^⁺, and total Fe levels in the LY294002 + ONSMP group were partially reduced compared with the LY294002-only group (Fig. 5C).

#### Enhancement of antioxidant capacity

Compared with the control group, H9C2 cells in the OGD group exhibited significantly impaired antioxidant capacity, as reflected by markedly reduced SOD content and CAT activity together with elevated ROS and H₂O₂ levels. These changes were reversed by ONSMP treatment relative to the OGD group. However, in LY294002-treated groups (with or without ONSMP), CAT activity was significantly lower than in the ONSMP group, whereas ROS and H₂O₂ levels were significantly higher. Notably, both LY294002-treated groups showed further increases in ROS and H₂O₂ levels compared with the OGD group (Fig. 5D and E). These findings indicate that ONSMP effectively enhances the antioxidant capacity of H9C2 cells and alleviates OGD-induced oxidative stress, and that this protective effect is markedly attenuated by AKT1 inhibition.

#### Improvement of mitochondrial morphology and function

Transmission electron microscopy showed that mitochondria in control H9C2 cells were plump, with well-organized cristae and uniform membrane density. After OGD treatment, mitochondria exhibited characteristic ferroptotic changes, including reduced volume, loss of cristae structure, and increased membrane density. ONSMP intervention markedly ameliorated these pathological alterations, restoring mitochondrial volume, partially reconstructing cristae, and decreasing membrane density. However, in the LY294002-treated groups (with or without ONSMP), mitochondrial damage was further exacerbated, with smaller organelles, complete loss of cristae, and higher membrane density (Fig. 5F). Consistently, mitochondrial membrane potential in the OGD group was significantly lower than in the control group and was significantly restored by ONSMP treatment, whereas membrane potential in the LY294002-treated groups was again significantly reduced (Fig. 5G). Collectively, these results indicate that ONSMP effectively inhibits ferroptosis by alleviating OGD-induced mitochondrial structural damage and functional impairment, and that this protective effect is markedly attenuated by AKT1 inhibition.

### Regulation of the cGMP-PKG signaling pathway by ONSMP in OGD-induced H9C2 cells

#### Upregulation of pathway protein interactions

**Fig. 6:**
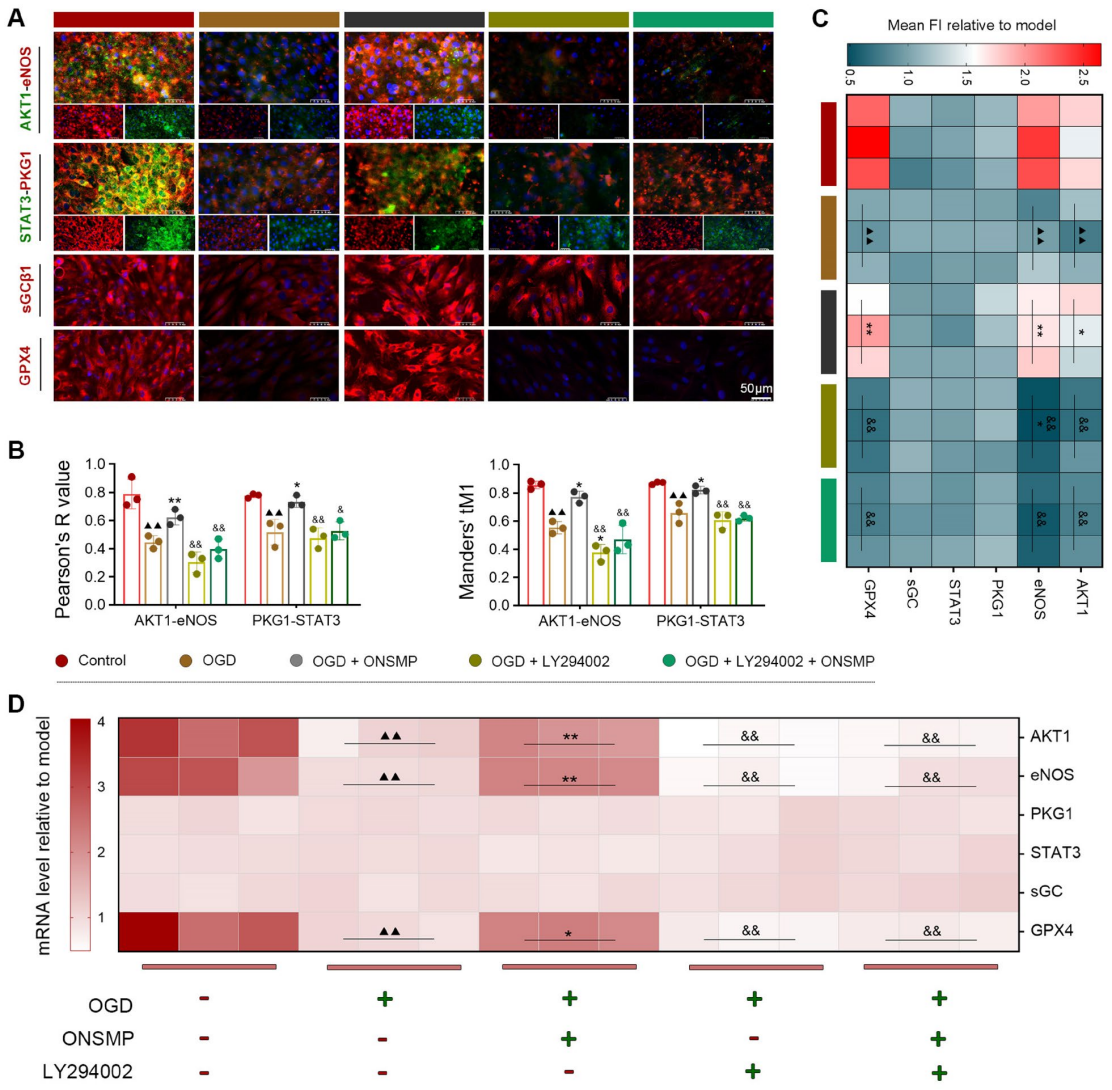
ONSMP enhances molecular interactions and protein/mRNA expression of the cGMP–PKG pathway in OGD-induced H9C2 cells. **A** IF detection of cGMP–PKG pathway proteins (× 400). **B** Pearson’s R and Manders’ tM1 colocalization analysis. **C** Quantification of mean FI for pathway proteins. **D** qRT-PCR analysis of mRNA expression of pathway components. (mean ± SD, n = 3). ns, not significant; ^▲▲^*P* < 0.01 vs. control group; ^*^P < 0.05, ^**^P < 0.01 vs. OGD group; ^&^*P* < 0.05, ^&&^*P* < 0.01 vs. ONSMP group

Pearson’s R and Manders’ tM1 analyses showed that in control H9C2 cells, AKT1–eNOS and PKG1–STAT3 exhibited strong colocalization, whereas OGD treatment significantly reduced colocalization of these pathway proteins. Following ONSMP treatment, colocalization of AKT1–eNOS and PKG1–STAT3 was significantly restored. In contrast, LY294002 treatment, whether combined with ONSMP or not, markedly weakened colocalization of these protein pairs. Notably, in the OGD + LY294002 group, the Manders’ tM1 value for AKT1–eNOS was further decreased compared with the OGD group (Fig. 6A and B). These results suggest that ONSMP may enhance interactions among key proteins in the cGMP–PKG signaling pathway by activating AKT1.

#### Upregulation of pathway protein and mRNA expression levels

IF and qRT-PCR analyses were performed to assess the effects of OGD injury and ONSMP treatment on the protein and mRNA expression of cGMP–PKG pathway components. At the protein level, the mean fluorescence intensity (FI) of AKT1, eNOS, and GPX4 was significantly reduced in the OGD group compared with the control group, and ONSMP treatment significantly restored their expression. In LY294002-treated groups—whether administered alone or in combination with ONSMP—the mean FI of these proteins was significantly lower than in the ONSMP group (Fig. 6A and C). The mRNA expression patterns were consistent with the protein-level findings: AKT1, eNOS, and GPX4 mRNA levels were significantly downregulated in the OGD group relative to the control, markedly upregulated by ONSMP treatment, and abolished by LY294002 co-treatment (Fig. 6D). These results indicate that ONSMP regulates the protein and mRNA expression of AKT1, eNOS, and GPX4 in the cGMP–PKG pathway via AKT1 activation. Notably, PKG1, STAT3, and sGCβ1 showed no significant differences among groups at either the protein or mRNA level, suggesting that ONSMP may modulate the activity of these downstream proteins primarily through post-translational mechanisms rather than altered expression.

#### Molecular activity and expression levels in the regulatory pathway

**Fig. 7:**
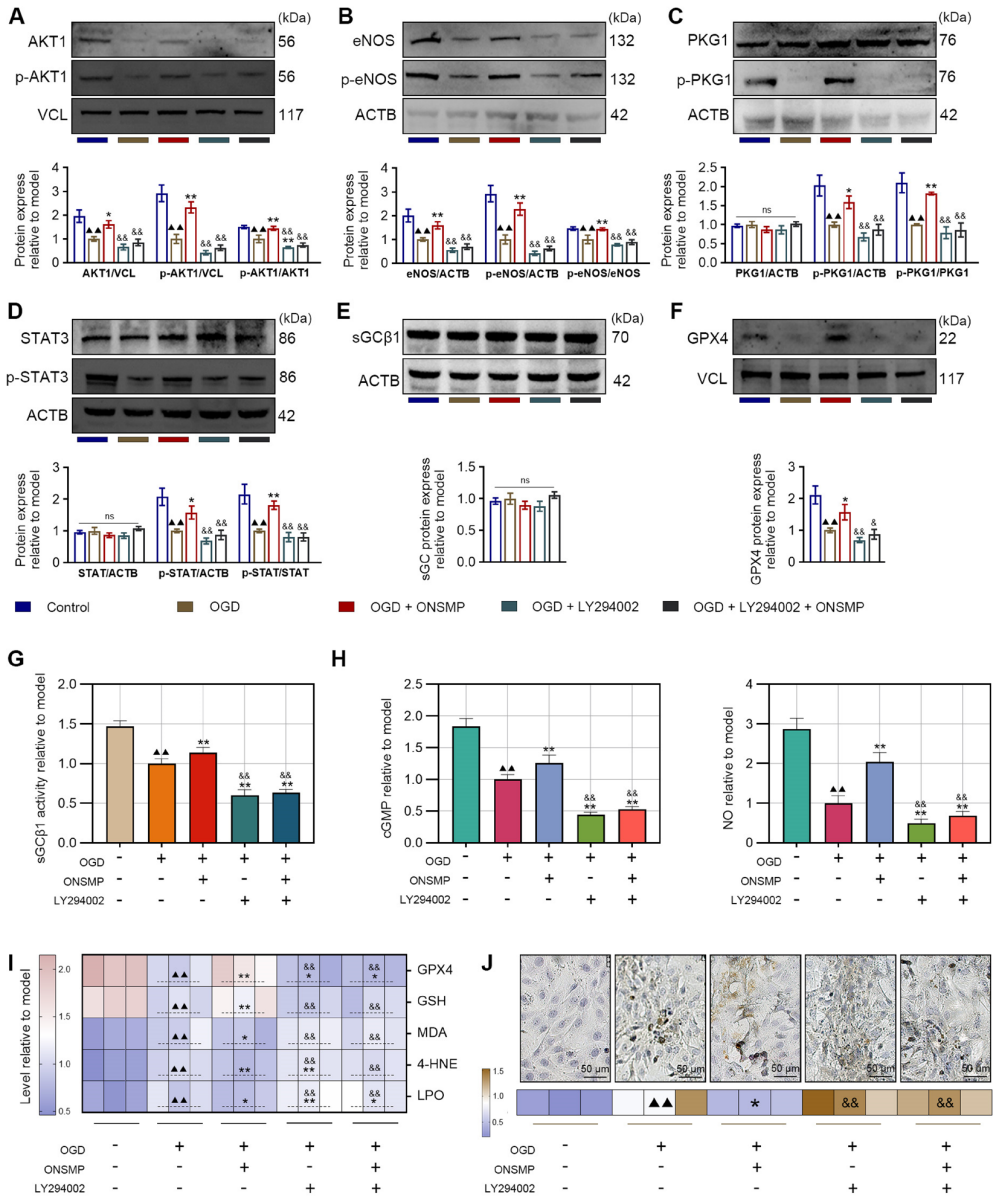
ONSMP regulates cGMP–PKG pathway activity and terminal effector molecules in OGD-induced H9C2 cells. **A**–**F** Western blot analysis of cGMP–PKG pathway proteins. **G** sGCβ1 activity assay. **H** Levels of pathway-related small molecules (cGMP and NO). **I** Levels of terminal effector molecules (GPX4, GSH, MDA, 4-HNE, and LPO). **J** IHC analysis of 4-HNE expression (× 400). (mean ± SD, n = 3). ^▲▲^*P* < 0.01 vs. control group; ^*^P < 0.05, ^**^P < 0.01 vs. OGD group; ^&^*P* < 0.05, ^&&^*P* < 0.01 vs. ONSMP group

Western blot analysis showed that, compared with the control group, the OGD group exhibited significant reductions in total protein levels of AKT1, eNOS, and GPX4, in their key phosphorylated forms (p-AKT1 and p-eNOS), and in the phosphorylation ratios p-AKT1/AKT1, p-eNOS/eNOS, p-PKG1/PKG1, and p-STAT3/STAT3; these changes were markedly restored by ONSMP treatment. However, LY294002 treatment—whether administered alone or in combination with ONSMP—significantly suppressed all of these indicators compared with the ONSMP group, and the p-AKT1/AKT1 ratio in the OGD + LY294002 group was even lower than in the OGD group (Fig. 7A–F). sGCβ enzyme activity was significantly decreased in the OGD group, substantially recovered by ONSMP, and again markedly reduced following LY294002 treatment (Fig. 7G). Similarly, levels of the downstream signaling molecules cGMP and NO were significantly diminished in the OGD group, effectively restored by ONSMP, but not fully reversed in LY294002-treated groups (with or without ONSMP), where they were further reduced below OGD levels (Fig. 7H). Collectively, this multidimensional evidence—from protein expression and phosphorylation status to key enzyme activity and downstream small-molecule signaling—demonstrates that ONSMP enhances the overall function of the cGMP–PKG signaling pathway primarily by promoting AKT1 activation.

#### Terminal effector levels of the regulatory pathway

GPX4, as the terminal antioxidant effector of the cGMP–PKG pathway, acts together with its essential cofactor GSH to suppress ferroptosis, whereas MDA, 4-HNE, and LPO are established markers of lipid peroxidation. Kit-based assays showed that, compared with the control group, the OGD group exhibited significantly decreased GPX4 and GSH levels and significantly increased MDA, 4-HNE, and LPO levels, and these alterations were markedly reversed by ONSMP treatment. Notably, in LY294002-treated groups—whether used alone or in combination with ONSMP—GPX4 and GSH levels were significantly lower, and MDA, 4-HNE, and LPO levels were significantly higher than in the ONSMP group, with all of these parameters further deteriorating compared with the OGD group (Fig. 7J). IHC analysis further confirmed the pattern of 4-HNE expression: 4-HNE was significantly upregulated in the OGD group relative to the control group, markedly reduced by ONSMP, and significantly increased again in both LY294002-treated groups compared with the ONSMP group (Fig. 7K). Collectively, these findings demonstrate that ONSMP inhibits ferroptosis by targeting AKT1, thereby restoring antioxidant system homeostasis and suppressing lipid peroxidation.

### ONSMP components modulate the cGMP–PKG pathway in OGD-injured H9C2 cells

#### Selection of representative active constituents of ONSMP

**Fig. 8:**
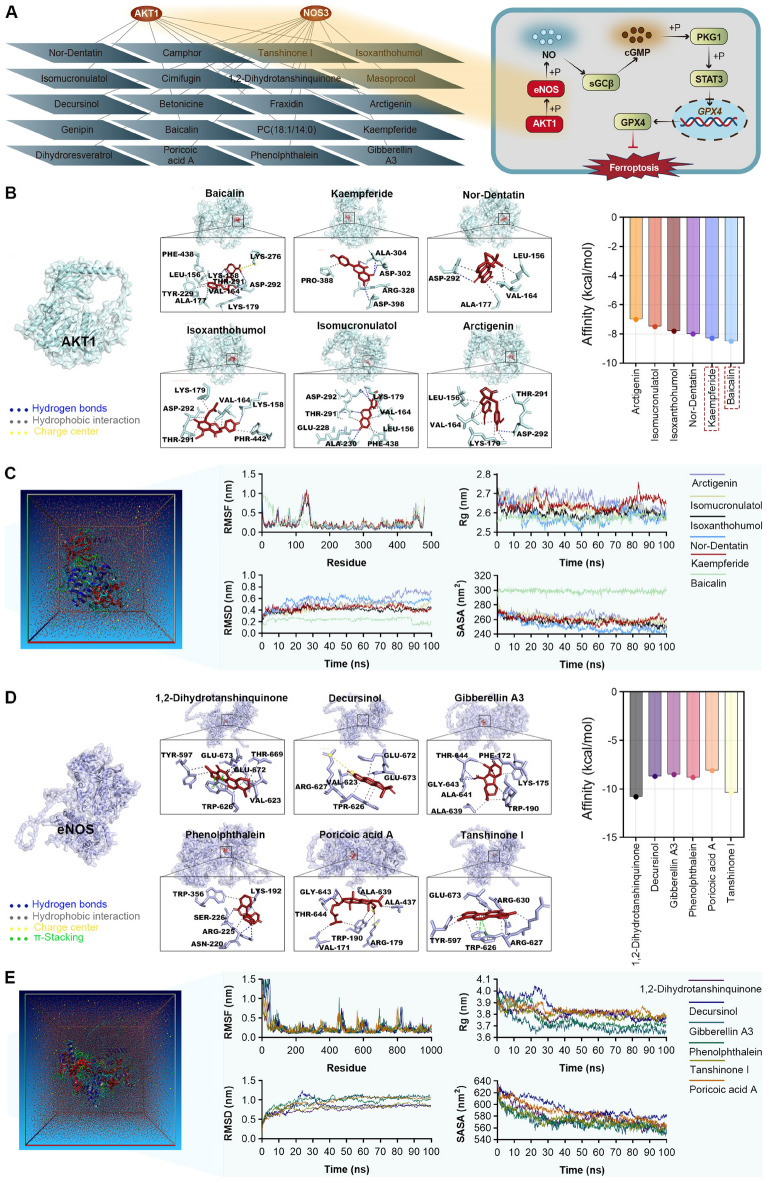
Molecular docking and dynamics simulations of AKT1, eNOS, and ONSMP active components. **A** Topological network analysis of AKT1, eNOS, and ONSMP active components. **B** Molecular docking of AKT1 with ONSMP active components. **C** Molecular dynamics simulations of AKT1–ONSMP active component complexes. **D** Molecular docking of eNOS with ONSMP active components. **E** Molecular dynamics simulations of eNOS–ONSMP active component complexes

To elucidate the material basis underlying ONSMP-mediated regulation of the cGMP–PKG pathway, AKT1—a central regulator in this pathway—was used as the target for reverse screening of representative active constituents of ONSMP. Topological network analysis revealed that AKT1 is associated with eight active ONSMP constituents (Fig. 8A). On this basis, molecular docking and molecular dynamics simulations were combined to identify the constituent with the most stable binding to AKT1, which was selected as a key candidate for subsequent mechanistic studies. To further refine the regulatory landscape of ONSMP on this pathway, the downstream effector eNOS was also analyzed, revealing that 11 active ONSMP constituents could potentially target eNOS (Fig. 8A). These findings support the notion that ONSMP activates the cGMP–PKG pathway through multi-component, multi-target synergistic modulation of the AKT1–eNOS axis.

- (1) AKT1 and active components of ONSMP
  Molecular docking between AKT1 and ONSMP active components showed that the top six compounds all had Affinity values below −7 kcal/mol, indicating strong binding. Interaction analysis revealed that Isomucronulatol formed five hydrogen bonds with AKT1 residues LYS-179A and GLU-228A and five hydrophobic contacts with LEU-156A and VAL-164A; Isoxanthohumol formed two hydrogen bonds with LYS-158A and ASP-292A and seven hydrophobic interactions with VAL-164A and LYS-179A; Arctigenin formed two hydrogen bonds with LYS-179A and ASP-292A and six hydrophobic interactions with LEU-156A and VAL-164A; Baicalin formed five hydrogen bonds with LYS-158A and LYS-179A, one salt bridge with LYS-276A, and seven hydrophobic interactions with LEU-156A; Kaempferide formed five hydrogen bonds with ASP-302A and GLY-303A; and Nor-Dentatin formed one hydrogen bond with ASP-292A and five hydrophobic interactions with LEU-156A (Fig. 8B).
  Molecular dynamics simulations further demonstrated that all six ONSMP active components formed highly stable and compact complexes with AKT1. RMSF analysis indicated higher flexibility at the N-terminus (residues 10–40) and C-terminus (last 20–30 residues; 0.42–0.88 nm), whereas residues within the catalytic domain/ATP-binding region (residues 50–450) showed markedly reduced fluctuations (0.10–0.35 nm), suggesting that each ligand stabilized the key structural framework by occupying active or allosteric sites. RMSD trajectories converged rapidly within 0–100 ns (rising from 0.05–0.30 nm to 0.20–0.70 nm and then remaining within a narrow range without sustained drift > 0.8 nm), indicating rapid attainment and maintenance of dynamic equilibrium. Rg (2.51–2.76 nm) and SASA (244–306 nm^2^) also stabilized within narrow windows after initial adjustments (Rg fluctuation < 0.2 nm, SASA fluctuation < 5%), consistent with tight protein folding, a stable hydrophobic core, and well-shielded surface residues. Notably, Nor-Dentatin extensively occupied the ATP-binding pocket, resulting in a slightly higher stable RMSD range (0.5–0.7 nm); Baicalin underwent local conformational adjustments at the binding interface but remained stably bound; and Kaempferide induced minimal fluctuations (0.10–0.30 nm) in residues near its binding site (80–100, 180–220, 350–480). These findings support differential yet highly efficient interaction modes between ONSMP components and AKT1 (Fig. 8C).
- (2) eNOS and active components of ONSMP
  Molecular docking between eNOS and ONSMP active components showed that the top six compounds all had Affinity values below −7 kcal/mol. Interaction analysis revealed that 1,2-Dihydrotanshinquinone formed one hydrogen bond with GLU-673A, eight hydrophobic interactions with TYR-597A and TRP-626A, and three π–stacking interactions with TRP-626A; Decursinol formed six hydrophobic interactions with VAL-623A and TRP-626A and one salt bridge with ARG-627A; Gibberellin A3 formed four hydrogen bonds with LYS-175A and ALA-641A and six hydrophobic interactions with PHE-172A and TRP-190A; Phenolphthalein formed three hydrogen bonds with ASN-220A and ARG-225A and four hydrophobic interactions with LYS-192A and TRP-356A; Poricoic acid A formed four hydrogen bonds with TRP-190A and GLY-643A, five hydrophobic interactions with VAL-171A and ALA-437A, and one salt bridge via ARG-179A; and Tanshinone I formed one hydrogen bond with ARG-630A, seven hydrophobic interactions with TYR-597A and TRP-626A, and two π–stacking interactions with TRP-626A (Fig. 8D).
  Molecular dynamics simulations demonstrated that all six ONSMP active components formed highly stable complexes with eNOS. RMSD trajectories reached equilibrium within nanoseconds and then fluctuated within a narrow range of 0.65–1.2 nm. Rg stabilized in a compact range of 3.72–4.05 nm, and SASA decreased by 5%–10% and remained stable (< 20 nm^2^), indicating rapid ligand-induced conformational convergence and tight interface packing. RMSF analysis showed high flexibility at the N-terminus (residues 1–50) and C-terminus (last 100–200 residues; 1.1–2.4 nm), whereas residues in the binding pocket and core functional domains (residues 50–850 and 300–600) exhibited markedly suppressed fluctuations (< 0.3 nm, some < 0.2 nm), consistent with deep ligand embedding and rigid stabilization of key regions. Specifically, Poricoic acid A induced a “high-RMSD steady state” in eNOS (0.9–1.1 nm); Tanshinone I showed a “flexible termini–rigid core” gradient; the Phenolphthalein binding interface was highly stable (RMSF 0.1–0.3 nm); Decursinol caused a moderate increase in flexibility near residues 1180–1203 (≈1.6 nm) that remained below the overall flexible regions; the Gibberellin A3 core region was highly convergent (RMSF < 0.2 nm); and 1,2-Dihydrotanshinquinone exhibited the greatest rigidity at the binding interface (RMSF < 0.3 nm). This multiscale dynamic analysis comprehensively delineates differential yet highly efficient regulatory modes of ONSMP components acting on eNOS (Fig. 8E).
  In summary, Baicalin and Kaempferide exhibited the most favorable combination of binding affinity and key interaction patterns in molecular docking and demonstrated excellent conformational stability and dynamic adaptability in molecular dynamics simulations. This “high affinity + high stability” profile makes them ideal candidate molecules for further investigation of ONSMP-mediated activation of the cGMP–PKG signaling pathway via AKT1, providing a solid basis for subsequent in vitro functional validation and mechanistic studies.

#### Amelioration of OGD-induced H9C2 cell injury and attenuation of ferroptosis

**Fig. 9:**
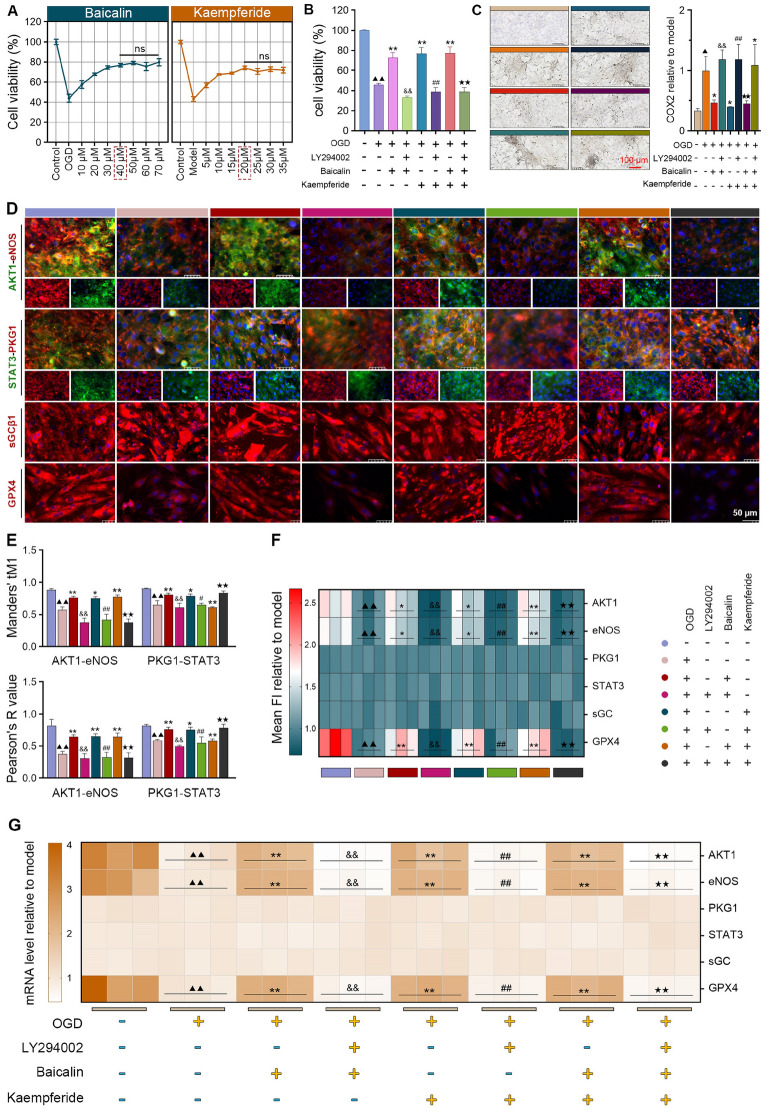
Regulation of the cGMP–PKG pathway by representative active components of ONSMP in OGD-induced H9C2 cells. **A** Determination of optimal concentrations of Baicalin and Kaempferide by CCK-8 assay. **B** Effects of Baicalin and Kaempferide on OGD-induced H9C2 cell viability. **C** IHC analysis of COX2 expression (× 400). **D** IF detection of cGMP–PKG pathway proteins (× 400). **E** Pearson’s R and Manders’ tM1 colocalization analysis. **F** Quantification of mean FI for pathway proteins. **G** qRT-PCR analysis of mRNA expression of pathway components. (mean ± SD, n = 3). ns, not significant; ^▲▲^*P* < 0.01 vs. control group; ^*^P < 0.05, ^**^P < 0.01 vs. OGD group; ^&&^*P* < 0.01 vs. Baicalin group; ^##^*P* < 0.01 vs. Kaempferide group; ^★★^*P* < 0.01 vs. Baicalin + Kaempferide group

To investigate the effects of the representative ONSMP active components Baicalin and Kaempferide on OGD-induced H9C2 cell injury and the AKT1-mediated cGMP-PKG signaling pathway, their effective intervention concentrations were first determined using a CCK-8 assay. The two compounds were dissolved in culture medium containing 10% FBS and serially diluted. Baicalin was tested at seven concentrations ranging from 10 to 70 μM; cell viability peaked in the 40–70 μM range with no significant differences among these groups (P > 0.05), and 40 μM was therefore selected as the optimal intervention concentration. Kaempferide was tested at seven concentrations ranging from 5 to 35 μM; the highest cell viability was observed in the 20–35 μM range, again with no significant intergroup differences (P > 0.05), and 20 μM was selected as the intervention concentration (Fig. 9A).

The protective effects of Baicalin and Kaempferide on OGD-induced H9C2 cell injury were further validated by CCK-8 assay and COX2 IHC staining. CCK-8 results showed that cell viability in the OGD group was significantly lower than in the control group, whereas treatment with Baicalin, Kaempferide, or their combination significantly restored cell viability. However, co-treatment with LY294002 markedly attenuated these protective effects (Fig. 9B). Consistently, COX2 IHC results indicated that COX2 expression was significantly higher in the OGD group than in the control group and was significantly reduced by Baicalin, Kaempferide, or combined treatment. The modulatory effects on COX2 expression were notably weakened when LY294002 was added (Fig. 9C). These results suggest that Baicalin and Kaempferide inhibit OGD-induced ferroptosis in H9C2 cells via an AKT1-dependent mechanism.

#### Upregulation of pathway protein interactions and protein/mRNA expression

Pearson’s R and Manders’ tM1 analyses showed that under OGD conditions, the colocalization of AKT1 with eNOS and of PKG1 with STAT3 was significantly reduced compared with the control group. Treatment with Baicalin, Kaempferide, or their combination markedly restored colocalization of these protein pairs, whereas the addition of LY294002 substantially weakened these effects (Fig. 9D, E). Further IF and qPCR analyses demonstrated that in the OGD group, the mean FI and mRNA expression levels of AKT1, eNOS, and GPX4 were significantly decreased. Baicalin, Kaempferide, or combined treatment effectively upregulated both the mean FI and mRNA levels of these proteins, whereas LY294002 significantly inhibited these effects. These findings indicate that the pathway-activating effects of Baicalin and Kaempferide are AKT1-dependent. Notably, no significant differences in mean FI or mRNA expression levels of PKG1, STAT3, and sGCβ1 were observed among the groups (Fig. 9D, F–G).

#### Regulation of cGMP-PKG pathway activity

**Fig. 10:**
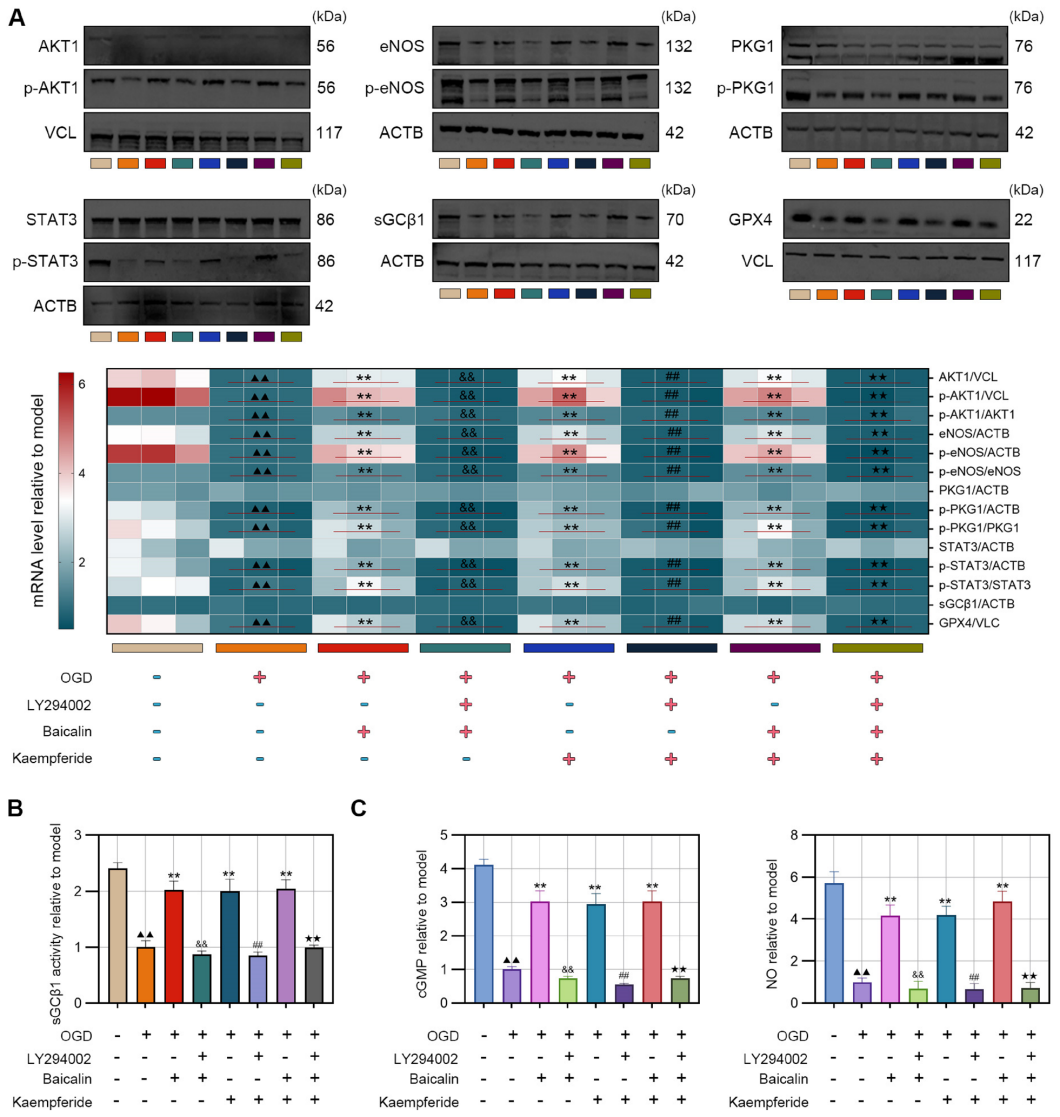
Regulation of the cGMP–PKG pathway by representative active components of ONSMP in OGD-induced H9C2 cells. **A** Western blot analysis of cGMP–PKG pathway proteins. **B** sGCβ1 enzymatic activity assay. **C** Levels of downstream small-molecule mediators cGMP and NO. (mean ± SD, n = 3). ^▲▲^*P* < 0.01 vs. control group; ^*^P < 0.05, ^**^P < 0.01 vs. OGD group; ^&&^*P* < 0.01 vs. Baicalin group; ^##^*P* < 0.01 vs. Kaempferide group; ^★★^*P* < 0.01 vs. Baicalin + Kaempferide group

Western blot analysis revealed that OGD treatment significantly downregulated the total protein levels of AKT1, eNOS, and GPX4, as well as their key phosphorylated forms (p-AKT1 and p-eNOS), and markedly reduced the phosphorylation ratios p-AKT1/AKT1, p-eNOS/eNOS, p-PKG1/PKG1, and p-STAT3/STAT3 compared with the control group. Treatment with Baicalin, Kaempferide, or their combination effectively restored the expression and phosphorylation status of these proteins, whereas LY294002 completely abolished the regulatory effects of these active compounds on pathway proteins (Fig. 10A). Further analysis showed that sGCβ1 enzymatic activity and the levels of the downstream signaling molecules cGMP and NO were significantly decreased in the OGD group relative to the control group and were significantly restored by Baicalin, Kaempferide, or combination treatment; however, LY294002 markedly reduced sGCβ1 activity and cGMP/NO levels compared with the drug-treated groups (Fig. 10B and C). Collectively, these findings demonstrate that Baicalin and Kaempferide—the representative bioactive components of ONSMP—exert their regulatory effects by activating AKT1, thereby enhancing the expression and functional activity of key molecules in the cGMP-PKG signaling pathway.

## Discussion

In the prevention and treatment of ischemic heart failure, contemporary Western medical approaches—primarily pharmacotherapy and interventional procedures—have effectively reduced mortality, but specific interventions that directly delay myocardial remodeling, the core pathological process, remain limited. In China, TCM, with its proven clinical efficacy and growing evidence base, has become an important component of comprehensive heart failure management [18]. Recent studies have shown that cardiomyocyte ferroptosis within the infarcted region, characterized by iron overload, ROS bursts, and lipid peroxidation, is a key driver of myocardial remodelling [19]. Our previous in vivo work demonstrated that ONSMP can inhibit this process; however, because of the cellular heterogeneity of myocardial tissue, the present study further employed an OGD-induced H9C2 cell model in vitro to elucidate the molecular mechanisms underlying the therapeutic effects of ONSMP.

Based on target prediction for blood-absorbed components, network pharmacology analysis, and core mechanistic screening, this study identified the cGMP–PKG signaling pathway as a central mechanism by which ONSMP inhibits ferroptosis in ischemic heart failure. This pathway is initiated by AKT1-mediated phosphorylation of eNOS at Ser1177, which enhances its catalytic activity and promotes the reaction of L-arginine with oxygen to generate NO and L-citrulline [26]. NO then binds to the ferrous heme (Fe^2^⁺) in the H-NOX domain of the sGCβ1 subunit, inducing a conformational shift from a resting to a highly active state and efficiently catalyzing the conversion of GTP into the second messenger cGMP [27]. The resulting increase in intracellular cGMP allows binding to two specific regulatory sites on PKG1, triggering conformational rearrangements that relieve autoinhibition of the catalytic core; subsequent autophosphorylation of multiple serine/threonine residues further enhances catalytic efficiency, substrate affinity, and dynamic subcellular localization, thereby establishing a positive feedback loop that maintains sustained activation. Activated PKG1 then interacts with STAT3 and promotes its phosphorylation [28]; phosphorylated STAT3 translocates to the nucleus, binds to DNA response elements in the GPX4 promoter, and drives its transcriptional expression [29, 30]. GPX4, in turn, uses two units of GSH as electron donors to maintain its antioxidant activity, reducing toxic PLOOH to non-toxic PLOH while oxidizing GSH to GSSG; GSSG is efficiently reduced back to GSH by glutathione reductase and NADPH, forming a complete antioxidant cycle that prevents pathological lipid peroxide accumulation and ultimately achieves precise inhibition of ferroptosis [31, 32].

Pharmacological studies showed that ONSMP markedly enhances the viability, migratory capacity, and survival of H9C2 cells subjected to OGD injury, indicating prominent anti-ferroptotic activity. Specifically, ONSMP downregulates the ferroptosis marker COX2, alleviates intracellular iron overload, and mitigates oxidative stress by increasing SOD content and CAT activity while reducing ROS and H₂O₂ accumulation. It also ameliorates typical ferroptosis-related mitochondrial abnormalities, including mitochondrial shrinkage, loss of cristae, and collapse of the mitochondrial membrane potential. At the molecular level, the protective effects of ONSMP are accompanied by upregulation of key proteins in the cGMP–PKG signaling pathway (AKT1, eNOS, sGCβ1, PKG1, and STAT3), strengthening of protein–protein interactions, and concomitant restoration of the pathway-related small molecules cGMP and NO. Importantly, AKT1, as the upstream core node of the cGMP–PKG pathway, is indispensable for these effects: treatment with the AKT1-specific inhibitor LY294002 completely abolishes the protective actions of ONSMP, directly supporting AKT1 as a primary target of ONSMP active components and establishing AKT1-dependent cGMP–PKG signaling as a central mechanism mediating precise ferroptosis regulation.

To elucidate the material basis of this mechanism, molecular docking and dynamics simulations identified Baicalin and Kaempferide as key active components with optimal binding efficiency and conformational stability toward AKT1. Functional assays showed that either compound alone or in combination effectively restored H9C2 cell viability under OGD injury and inhibited the OGD-induced upregulation of the ferroptosis marker COX2. At the level of molecular regulation, Baicalin and Kaempferide significantly increased the protein expression, activity, and mRNA levels of AKT1, eNOS, and GPX4, as well as the interactions between AKT1–eNOS and PKG1–STAT3, which were suppressed under OGD conditions. Although the transcriptional levels of PKG1, STAT3, and sGCβ1 were not significantly altered, their phosphorylation (PKG1, STAT3) and enzymatic activity (sGCβ1) were effectively enhanced. Furthermore, these compounds promoted the generation of the downstream second messengers cGMP and NO via AKT1 activation. Collectively, these findings indicate that Baicalin and Kaempferide are core active constituents of ONSMP that modulate the cGMP–PKG signaling pathway.

Existing literature further supports these findings. Previous studies have shown that Baicalin, a key component of ONSMP, can specifically bind to the pleckstrin homology domain of AKT1, activating AKT1 by inhibiting its membrane translocation and promoting phosphorylation at Ser473, thereby enhancing eNOS activity and NO production and ultimately alleviating myocardial injury [33–35]. Another important component, Kaempferide, similarly upregulates AKT1 phosphorylation and confers cardioprotection by suppressing inflammatory cytokine release, improving oxidative stress (as evidenced by decreased MDA and ROS levels and increased SOD activity), and correcting dysregulated glucose and lipid metabolism [36, 37]. Although the present study did not systematically investigate the mechanisms of other constituents, previous reports indicate that Isoxanthohumol [38, 39] and Arctigenin [38, 39] can also activate the AKT/eNOS signaling pathway, enhance eNOS activity, reduce lipid accumulation and oxidative stress, and/or promote nuclear translocation of Nrf2 and HO-1 gene expression via antioxidant response elements. Collectively, this body of evidence supports the scientific plausibility that active components of ONSMP exert cardioprotective effects at least in part by targeting the AKT1–eNOS–cGMP–PKG1 signaling pathway.

**Fig. 11:**
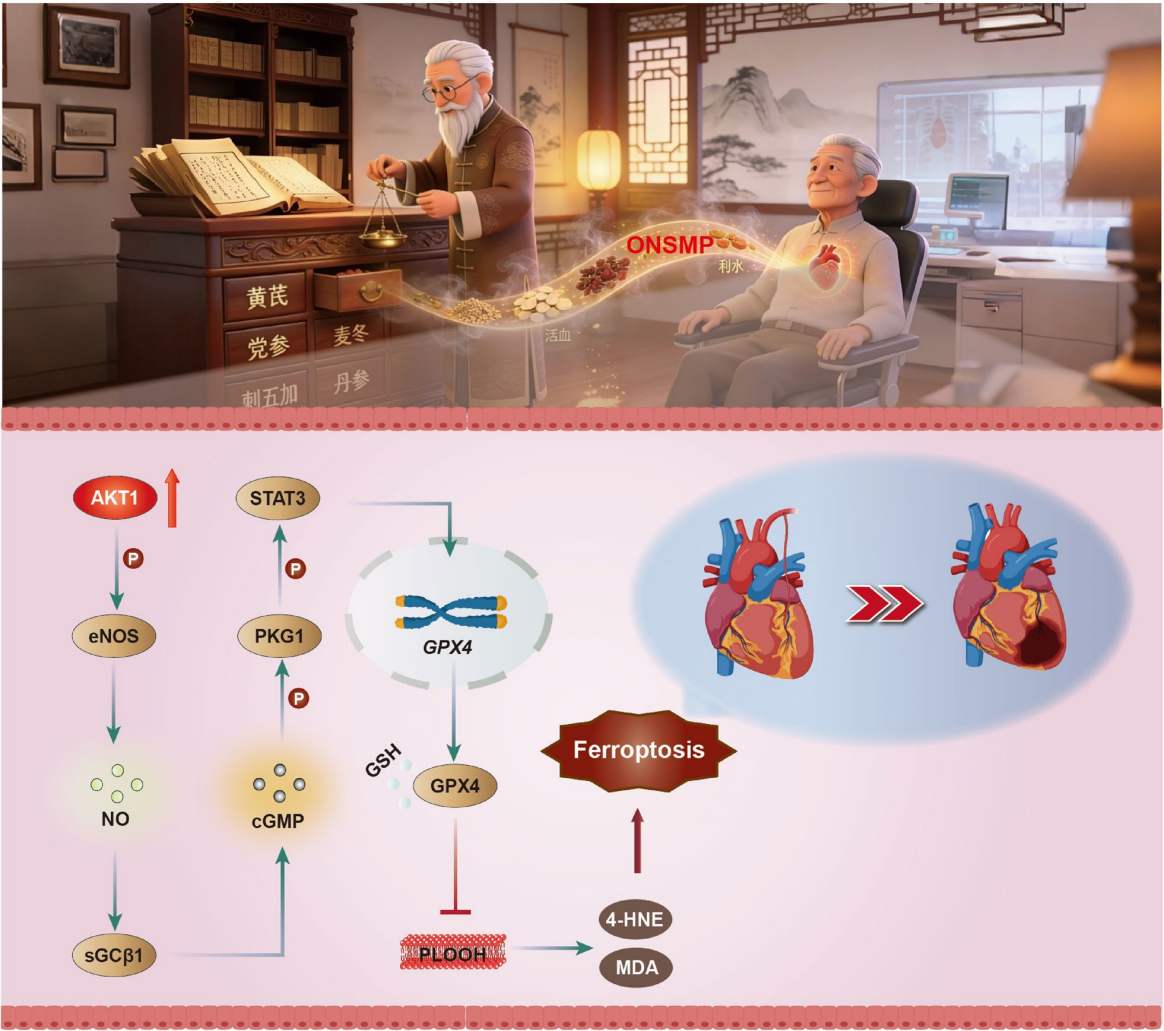
ONSMP activates the cGMP–PKG signaling pathway to inhibit cardiomyocyte ferroptosis. ONSMP activates AKT1, thereby regulating the cGMP–PKG signaling pathway and promoting GPX4 expression. GPX4 then uses GSH as an electron donor to maintain its antioxidant activity, effectively eliminating lipid peroxides and suppressing ferroptosis in cardiomyocytes

In summary, this study systematically demonstrates that ONSMP activates AKT1, which in turn drives multidimensional activation of the cGMP–PKG signaling pathway—including protein–protein interactions, modulation of enzyme activity, post-translational modifications, small-molecule signaling, and transcriptional regulation—ultimately leading to effective inhibition of ferroptosis (Fig. 11). However, several limitations and related scientific questions remain to be addressed. (1) Computational simulations suggested that ONSMP active components can also bind eNOS, but experimental data showed that downstream pathway activation was ineffective when AKT1 was inhibited. This discrepancy may reflect the extensive downstream network of AKT1: its inhibition likely triggers complex cascading effects, severely impairing cell viability and downregulating eNOS expression, thereby creating a vicious cycle of insufficient eNOS protein and blocked pathway activation. This phenomenon was particularly pronounced in the OGD plus AKT1 inhibitor group and could not be reversed by ONSMP treatment, indicating that future studies should employ eNOS-specific inhibition strategies to independently assess its function. (2) ONSMP markedly reduces free iron levels during ferroptosis inhibition, an effect that may not be solely attributable to global pathway activation and may also involve direct regulation of iron metabolism–related genes. This potential mechanism represents an important direction for future research to fully elucidate the molecular basis of the anti-ferroptotic actions of ONSMP. (3) Although the H9C2 cell line offers advantages such as stable availability, well-established culture protocols, and favorable reproducibility—making it suitable for preliminary mechanistic screening and initial validation of key causal pathways—it still differs from mature cardiomyocytes in differentiation status, energy metabolic profile, mitochondrial oxidative phosphorylation capacity, and stress response patterns [40, 41]. These differences may influence the manifestation of ferroptosis-related phenotypes and the magnitude and temporal dynamics of cGMP–PKG signaling responses. Consequently, findings derived from H9C2 cells in this study should be interpreted as supportive evidence and mechanistic clues for in vivo observations, rather than as directly equivalent to the physiological state of native cardiomyocytes. It is important to emphasize that the pivotal mechanisms proposed here have not yet been directly validated at the animal tissue level, which is a notable limitation of the present work. Future investigations should systematically and directly verify the regulatory relationship between ferroptosis and the cGMP–PKG signaling axis identified in this study through in vivo or ex vivo experiments using animal myocardial tissues. In parallel, critical parameters—including ferroptosis phenotypes, GPX4 functional status, and activation kinetics of the cGMP–PKG signaling axis—should be re-evaluated in cellular models with higher physiological fidelity, such as primary cardiomyocytes or iPSC-derived cardiomyocytes. Moreover, more sophisticated in vitro systems (e.g., combined hypoxia/nutrient deprivation stress, inflammatory cytokine stimulation, or 3D culture models) could be established to corroborate the proposed signaling framework from multiple experimental dimensions. Through comparative integration of data from animal tissues and other physiologically relevant models, the robustness and scope of the mechanistic conclusions can be more rigorously defined, while explicitly acknowledging the inherent constraints of the H9C2 model system.

## Conclusion

ONSMP effectively ameliorates OGD-induced ferroptosis in H9C2 cells. One mechanism involves active components such as Baicalin and Kaempferide, which promote AKT1 phosphorylation, thereby activating the cGMP–PKG signaling pathway and upregulating GPX4 expression. GPX4 then uses GSH as an electron donor to maintain its antioxidant activity and effectively eliminate the accumulation of lipid peroxides.

## Supplementary Information

Supplementary Material 1. Table S1. Comprehensive list of compounds.

Supplementary Material 2. Table S2. ONSMP blood-entering components.

Supplementary Material 3. Table S3. Targets of ONSMP components.

Supplementary Material 4. Table S4. Ischemic heart failure-related proteins.

Supplementary Material 5. Table S5. Ferroptosis-related proteins

## Data Availability

The datasets used or analyzed throughout this study are available from the corresponding author upon reasonable request.

## Abbreviations

4-HNE: 4-Hydroxynonenal
ACTB: Beta-actin
AKT: Protein kinase B
CAT: Catalase
cGMP: Cyclic guanosine monophosphate
eNOS: Endothelial nitric oxide synthase
Fe: Iron
FBS: Fetal bovine serum
GPX4: Glutathione peroxidase 4
GSH: Glutathione
H₂O₂: Hydrogen peroxide
IF: Immunofluorescence
IHC: Immunohistochemistry
LPO: Lipid peroxide
MDA: Malondialdehyde
NO: Nitric oxide
OGD: Oxygen–glucose deprivation
ONSMP: Optimized New Shengmai Powder
p-: Phospho-
PB: Prussian blue
PKG: Protein kinase G
ROS: Reactive oxygen species
sGC: Soluble guanylate cyclase
SOD: Superoxide dismutase
STAT3: Signal transducer and activator of transcription 3
TCM: Traditional Chinese medicine
VCL: Vinculin
